# Supplementary material for: Study protocol for a randomised controlled trial assessing the clinical and cost-effectiveness of the Journeying through Dementia (JtD) intervention compared to usual care
Source: BMJ Open. 2019 Sep 13;9(9):e029207. doi: 10.1136/bmjopen-2019-029207 (PMC6747651; doi:10.1136/bmjopen-2019-029207)
Supplement: Author's manuscript [file bmjopen-2019-029207.draft_revisions.pdf]

BMJ Open is committed to open peer review. As part of this commitment we make the peer review history of every article we publish publicly available.

When an article is published we post the peer reviewers' comments and the authors' responses online. We also post the versions of the paper that were used during peer review. These are the versions that the peer review comments apply to.

The versions of the paper that follow are the versions that were submitted during the peer review process. They are not the versions of record or the final published versions. They should not be cited or distributed as the published version of this manuscript.

BMJ Open is an open access journal and the full, final, typeset and author-corrected version of record of the manuscript is available on our site with no access controls, subscription charges or pay-per-view fees (http://bmjopen.bmj.com).

If you have any questions on BMJ Open's open peer review process please email info.bmjopen@bmj.com

BMJ Open

A study protocol for a randomised controlled trial of the clinical and cost-effectiveness of the Journeying through Dementia (JtD) intervention compared to usual care

Journal:	*BMJ Open*
Manuscript ID	bmjopen-2019-029207
Article Type:	Protocol
Date Submitted by the Author:	18-Jan-2019

Complete List of Authors:	Wright, Jessica; University of Sheffield, Clinical Trials Research Unit<br>Foster, Alexis; University of Sheffield, School of Health and Related Research<br>Cooper, Cindy; The University of Sheffield, Sheffield Clinical Trials Research Unit, School of Health and Related Research<br>Sprange, Kirsty; The University of Nottingham, Nottingham Clinical Trials Research Unit<br>Walters, Stephen; University of Sheffield, ScHARR<br>Berry, Katherine; The University of Manchester, School of Psychological Sciences<br>Moniz-Cook , Esme ; The University of Hull, School of Health and Social Work<br>Loban, Amanda; University of Sheffield, School of Health and Related Research<br>Young, Tracey; The University of Sheffield, School of Health and Related Research<br>Craig, Claire; Sheffield Hallam University<br>Dening, Tom; University of Nottingham, Division of Psychiatry & Applied Psychology, School of Medicine<br>Lee, Ellen; University of Sheffield, Clinical Trials Research Unit<br>Beresford-Dent, Julie; University of Bradford, Centre for Applied Dementia Studies<br>Thompson, Benjamin; The University of Sheffield, School of Health and Related Research<br>Young, Emma; The University of Sheffield, School of Health and Related Research<br>Thomas, Benjamin; The University of Sheffield, School of Health and Related Research<br>Mountain, Gail; University of Bradford, Centre for Applied Dementia Studies

Keywords:	Randomised Controlled Trial, Dementia < NEUROLOGY, Research Protocol, Post diagnostic support, Self-management, Well-being

SCHOLARONE™
Manuscripts

TITLE PAGE

Title

A study protocol for a randomised controlled trial of the clinical and cost-effectiveness of the Journeying through Dementia (JtD) intervention compared to usual care

Corresponding author

Jessica Wright
Journeying Through Dementia (JtD) Trial,
Sheffield Clinical Trials Research Unit,
ScHARR, The University of Sheffield,
Regent's Court,
30 Regent St, Sheffield S1 4DA
Telephone: 0114 222 4304
Email: Jessica.wright@sheffield.ac.uk

Author Names/Affiliations

Jessica Wright, Sheffield Clinical Trials Research Unit, School of Health and Related Research, The University of Sheffield, Sheffield, UK

Alexis Foster, School of Health and Related Research, The University of Sheffield, Sheffield, UK

Cindy Cooper, Sheffield Clinical Trials Research Unit, School of Health and Related Research, The University of Sheffield, Sheffield, UK

Kirsty Sprange, Nottingham Clinical Trials Research Unit, The University of Nottingham, Nottingham, UK

Stephen Walters, School of Health and Related Research, The University of Sheffield, Sheffield, UK

Katherine Berry, Manchester Institute for Collaborative Research on Ageing, The University of Manchester, Manchester, UK

Esme Moniz-Cook, School of Health and Social Work, The University of Hull, Hull, UK

Amanda Loban, Sheffield Clinical Trials Research Unit, School of Health and Related Research, The University of Sheffield, Sheffield, UK

Tracey Young, School of Health and Related Research, The University of Sheffield, Sheffield, UK

Claire Craig, Art & Design Research Centre, Sheffield Hallam University, Sheffield, UK

Tom Dening, Institute of Mental Health, The University of Nottingham, Nottingham, UK

Ellen Lee, Sheffield Clinical Trials Research Unit, School of Health and Related Research, The University of Sheffield, Sheffield, UK

For peer review only

Julie Beresford-Dent, Centre for Applied Dementia Studies, University of Bradford, Bradford, UK

Benjamin John Thompson, Sheffield Clinical Trials Research Unit, School of Health and Related Research, The University of Sheffield, Sheffield, UK

Emma Louise Young, Sheffield Clinical Trials Research Unit, School of Health and Related Research, The University of Sheffield, Sheffield, UK

Benjamin David Thomas, Sheffield Clinical Trials Research Unit, School of Health and Related Research, The University of Sheffield, Sheffield, UK

Gail Mountain, Centre for Applied Dementia Studies, University of Bradford, Bradford, UK

Word Count: 5709 (excluding references, abstract, tables and figures)

For peer review only

For peer review only - http://bmjopen.bmj.com/site/about/guidelines.xhtml

ABSTRACT

Introduction

UK Memory Services are being encouraged to provide post diagnostic treatment to those with dementia but the availability of evidence-based interventions following diagnosis has not kept pace with increase in demand. To address this, Journeying through Dementia (JtD) was developed. A randomised controlled trial (RCT), based on a pilot study, is in progress.

Methods and analysis

The RCT is a pragmatic, two-arm, parallel group trial designed to test the clinical and cost-effectiveness of JtD compared to usual care. Recruitment will be through NHS services, third sector organisations and Join Dementia Research. The sample size is 486 randomised (243 to usual care and 243 to the intervention in addition to usual care). Participants can choose to have a supporter involved. The primary outcome measure for participants is the Dementia Related Quality of Life measure collected at baseline and at follow-up 8 months' post randomisation. Secondary outcome measures will be collected from participants and supporters at those visits. Participants will also be followed-up at 12 months' post randomisation. Embedded qualitative and fidelity sub-studies will be conducted.

Analyses will compare the two arms of the trial on an intention to treat as allocated basis. The primary analyses will compare the mean DEMQOL scores of the participants with dementia at 8 months between the two study arms. A cost-effectiveness analysis will be undertaken of the incremental cost per Quality Adjusted Life Years of the JtD intervention compared with usual care. Qualitative and fidelity sub-studies will be analysed through framework analysis and fidelity assessment tools respectively.

Ethics and dissemination

Research Ethics Committee and Health Research Authority approval have been obtained. A Data Monitoring and Ethics Committee has been constituted. Dissemination will be via publications, conferences, websites and social media. Finalised intervention materials will be offered via open access.

Trial registration number

ISRCTN17993825

For peer review only

For peer review only - http://bmjopen.bmj.com/site/about/guidelines.xhtml

STRENGTHS AND LIMITATIONS OF THE STUDY

- People living with dementia were involved in developing the content of the Journeying through Dementia (JtD) intervention and are involved in advising on the study.
- The JtD intervention includes sessions without supporters present, to help develop independence and confidence, and is one of the only interventions that people with dementia can participate in without supporters.
- The JtD study will recruit up to 500 participants and is therefore one of the largest dementia related trials in the UK.
- A qualitative sub-study will explore the mediators and moderators of the intervention.
- Recruitment can be challenging in this population but we plan to try multiple pathways for recruitment including through services, the Join Dementia Research database, promotion and the third sector.

INTRODUCTION

The impact on the economy, for services and for individuals living with dementia and their family carers is larger for dementia than for all other long-term illnesses in people aged 60 and over.[1] Two thirds of people with dementia live in the community, and half of these require some form of support.[2] As a result, dementia research (both for cure and for care) in the NHS and social care is important, and this is reflected in UK health policy.[3] In 2009, the UK Government announced a National Dementia Strategy, which mandated the establishment of memory services and aimed to increase the rates of early diagnosis and improve support for people in the early stages of dementia.[4]

The National Audit of Memory Services (2013) found there had been a fourfold increase in numbers presenting since 2010/11, and in 2013 49.3% were in the early stages of the condition.[5] Earlier diagnosis allows individuals to receive treatment earlier and enables both the individual and memory services to plan more effectively for the future.[6] Memory services have been strongly encouraged to provide post diagnostic treatment and support,[7,8] but the availability of appropriate evidence-based interventions has not kept pace with the increase in demand, in particular for those with early stages of dementia. The lack of appropriate interventions has led to inconsistency between Trusts as to what is being offered to people post diagnosis.

The potential value of psychosocial interventions for people in the early stages of dementia is recognised[9–12] and is also driven by the knowledge that a cure for dementia is unlikely in the near future. Psychosocial interventions are diverse but their common theme is that they do not involve the use of medication and instead focus on supporting people to overcome challenges and maintain independence and well-being. However, whilst there has been some shift, the use of psychosocial interventions within dementia care has been a neglected area in both research and practice.[11]

There is a growing body of evidence to demonstrate how individuals with dementia can be supported to use self-management based techniques (sometimes in combination with other interventions such as cognitive rehabilitation and occupational therapy).[13–18] A qualitative study which interviewed people with dementia who attended a self-management programme reported that participants identified the opportunity for peer support as a benefit and felt the programme

For peer review only

could be improved by having more emphasis on maintaining activities and relationships and improving positive wellbeing.[19] The Healthbridge evaluation[20] and the Mental Health Foundation evaluation[21] found evidence that people with dementia and their carers also benefit from receiving group based peer support.

The Journeying through Dementia (JtD) intervention was developed from the Lifestyle Matters programme and a pilot study was conducted to examine the feasibility of a future population-based larger trial of this intervention.[22] The intervention was found to be acceptable to both people with dementia and their supporters. Reported benefits included increased confidence and self-efficacy, engagement in activities and re-engagement with fun and friendships.[22] The intervention is manualised, based on occupational therapy principles, and is designed to support independence and well-being. The intervention incorporates elements of self-management and group-based peer support and has been designed to improve the quality of life for people in the early stages of dementia.

The JtD randomised controlled trial (RCT) will test the clinical and cost effectiveness of the JtD intervention. Funding was obtained through the National Institute of Health Research (NIHR) Health Technology Assessment (HTA) theme to conduct the RCT. This paper describes the research protocol for undertaking the RCT which started recruitment in November 2016.

AIMS AND OBJECTIVES

The primary aim of the JtD trial is: to determine the clinical and cost-effectiveness of the JtD intervention for people in the early stages of dementia. To meet this aim, the objectives are to:

1. Undertake a pragmatic RCT evaluating the clinical and cost-effectiveness of the JtD intervention.
2. Conduct fidelity checks regarding the delivery of the JtD intervention.
3. Undertake an embedded qualitative sub-study to explore the mechanisms that might result in intervention effectiveness
4. Identify how the intervention might be realistically delivered through services.
5. Be an exemplar of best practice in involvement of people living with dementia in a large trial.

METHODS

Trial design

The JtD trial is a pragmatic, two-arm, parallel group, individually randomised RCT. It uses a superiority framework to deliver an intention-to-treat comparison of the JtD intervention with usual care.

The trial includes three sub-studies. The first is a health economics evaluation using a cost-effectiveness analysis of the incremental cost per Quality Adjusted Life Year (QALY) of the JtD intervention compared with usual care.

The second sub-study is a fidelity assessment, using an intervention fidelity framework based on that identified by the Behaviour Change Consortium and National Institute for Health and Care Excellence (NICE).[23,24]

For peer review only - http://bmjopen.bmj.com/site/about/guidelines.xhtml

The third sub-study is a qualitative study, in line with Medical Research Council (MRC) guidance on developing and evaluating complex interventions.[25] The qualitative sub-study will involve semi-structured interviews with participants, participating supporters, facilitators and supervisors.

Randomisation and blinding

To minimise bias, allocation will be concealed through the use of a centralised web-based randomisation service. The randomisation sequence will be stratified by delivery site and constrained by a fixed block size to ensure participants are allocated evenly to each arm of the trial at each delivery site. Participants will be randomised in equal numbers to intervention and control arms. An unblind member of the research team who will not be conducting outcome assessments will enter the participants' details into the randomisation system and inform the relevant parties of the outcome.

Members of the Trial Steering Committee (TSC), study statisticians, health economists and outcome assessors will be blinded to treatment allocation whilst the trial is ongoing. For practical reasons, some members of the research team will not be blinded, including the Trial Manager and Chief Investigator. Due to the nature of the intervention, participants will not be blinded. If the outcome assessors know (or suspect) they have been unblinded this will be recorded on an unblinding form.

Participants

Persons with early-stage dementia will be approached to take part in the trial. A family member, friend or neighbour that provides support to the study participant (referred to as the 'participating supporter') can be approached to take part in the trial, but only if invited to do so by the person with dementia. Participating supporters will automatically be randomised to the same arm as the participant. See Figure 1 for participant flow through the study.

(Insert Figure 1 here)

Eligibility criteria

Participants will be eligible for the study if they:

1. have a diagnosis of any form of dementia,
2. have a Mini Mental State Examination (MMSE) score of 18 or more, taken less than 2 months' pre-consent,
3. have capacity to make informed decisions,
4. are living in the community in their own or sheltered accommodation (those living in residential or nursing care are not eligible),
5. are willing to attend the JtD intervention,
6. are able to converse and communicate in English, and
7. are not taking part in any other pharmacological or psychosocial intervention studies at the time of enrolment.

Participating supporters will be eligible if they are aged 18 years or over, and are named by the person with dementia as their supporter, the person with dementia wishes them to take part, they can converse and communicate in English, and have the ability to give informed consent.

For peer review only - http://bmjopen.bmj.com/site/about/guidelines.xhtml

Site selection and participant recruitment

The JtD trial will operate in England within 13 NHS trusts with specialist dementia services. The sites will be selected based on a convenience sample of locations clustered geographically around the north and midlands (for a list of sites, see study ISRCTN webpage[26]).

Previous studies involving persons with dementia have shown that recruiting participants to such studies can be challenging.[27] To ensure sufficient numbers are recruited to the study a number of recruitment pathways will be used: referral from clinical teams; mail-outs to eligible patients via primary or secondary care clinical teams; study promotion via posters etc.; third sector organisations; and the Join Dementia Research database. The research team will contact interested participants who agree to be approached and send further information about the study. A consent and eligibility visit will be arranged if they would like to take part where trained researchers will assess eligibility and request written informed consent.

Intervention

JtD is a manualised intervention consisting of twelve weekly facilitated groups with 8-12 participants with dementia, which take place over successive weeks. Each participant also receives four one-to-one sessions with one of the intervention facilitators, to pursue individual goals. The first one-to-one session takes place before the start of the group intervention, with the remaining three being scheduled during, or shortly after, the 12 weeks and at a location and time agreed with the participant. The group aspect of intervention should be delivered in a community venue.

The content of the JtD intervention is summarised below:

a. Ways of thinking about dementia (what is dementia, effects on everyday life, challenging stereotypes, sharing coping strategies).
b. Keeping physically well (relationship between physical and mental wellbeing, embedding health activity in everyday life, diet).
c. Memory (strategies to aid memory, impact on everyday life and learning and practicing new techniques).
d. Keeping mentally well (relationships between anxiety and memory, and dementia and stress).
e. Endings (celebration of achievements and how to move forward).

Participants are encouraged to select different topics from the manual and explore them with guidance and suggestions from facilitators. Participants are also able to suggest topics not within the manual. One essential component of the intervention is the enactment of activities, particularly in the community; three of the twelve group meetings should be out of venue activities. Participants are able to invite a supporter to participate in the group aspect of the intervention during sessions 1, 6 and 12, and in the individual sessions if the participant finds this helpful in achieving their goals.

Facilitating staff

The intervention should be facilitated by a minimum of two NHS staff who are experienced with working with people with dementia. Facilitators will normally be someone employed on Agenda for Change Bands 3-5. Facilitators must receive a two-day training course prior to delivering the

For peer review only

intervention. In some cases, for example they are a reserve facilitator, they may receive a shortened course supported by online resources created for this purpose. Facilitators will be supervised by a colleague experienced in supervision who has also attended the facilitator training and is of suitable seniority and experience. The supervisors themselves will be supervised by a member of the central study team who is a Clinical Psychologist and is experienced in the 'train the trainer' method[28].

Patient and public involvement (PPI)

People living with dementia were involved in developing the content of JtD[29] and in the feasibility study.[22] The JtD Trial Steering Committee will include a member who is living with dementia. Additionally, an advisory group (experts by experience) will meet at intervals throughout to provide input into study materials used by participants such as information sheets and interview guides. We also plan to involve people living with dementia in some aspects of the qualitative data analysis and in developing and delivering the study dissemination plans.

OUTCOMES

The primary outcome is the Dementia Related Quality of Life Measure (DEMQOL)[30,31] at 8 months' post randomisation.

The secondary outcomes are:

- European Quality of Life - 5 Dimensions (EQ-5D-5L)[32,33]
- Patient Health Questionnaire-9 (PHQ-9)[34,35]
- Generalised Anxiety Disorder-7 (GAD-7)[36]
- General Self-Efficacy Scale (GSE)[37]
- Diener's Flourishing Scale (DFS)[38]
- Self-Management Ability Scale (SMAS)[39]
- Instrumental Activities of Daily Living (IADL)[40]
- Health and Social Care Resource Use Questionnaire (HSCRU)[22]
- Sense of Competency Questionnaire (SCQ)[41,42]

The outcome measures used were selected to measure the following key components of the intervention: mental wellbeing or mood (DEMQOL, PHQ-9, GAD-7); building relationships and a sense of connectedness (DFS, SMAS); belief that life is meaningful despite dementia (DEMQOL, GSE, DFS, SMAS); and Instrumental Activities of Daily Living (IADL) and strategies to maintain cognitive functioning (IADL). Additionally, they also support analysis of the cost-effectiveness of the intervention (DEMQOL, EQ-5D-5L, HSCRU) and the participating supporters' perceptions of competence (SCQ). Dementia specific outcome measures are those recommended for use across Europe.[43] Non dementia specific outcome measures were selected if there was no appropriate dementia specific measure available.

DATA COLLECTION

Quantitative

Data will be collected from all participants living with dementia at eligibility/consent, baseline, 8 and 12 months' post randomisation and from participating supporters at baseline and 8 months' post

For peer review only - http://bmjopen.bmj.com/site/about/guidelines.xhtml

randomisation (see Table 1). The outcome measures will be interviewer-administered at face-to-face visits by blinded outcome assessors who have received training to deliver the measures to people living with dementia and their supporters. The follow-up outcome measures will be collected 2 weeks pre and 8 weeks post the date they are due. Participant retention will be promoted by regular communication with the participants and supporters through newsletters and Christmas cards.

Table 1 Outcome measures and time-points for collection. * Denotes the primary outcome measure.

Measure	**Participant**				**Participating Supporter**	
	**Eligibility and Consent visit**	**Baseline** <2 months prior to the intervention start date	**8 Months** Due < 2 weeks pre and < 8 weeks post randomisation	**12 Months** Due < 2 weeks pre and < 8 weeks post randomisation if within study timelines	**Baseline** Due <2 months prior to the intervention	**8 Months** Due < 2 weeks pre and < 8 weeks post randomisation
Capacity assessment	✓					
Mini Mental State Examination	✓					
Eligibility checklist	✓				✓	
Baseline demographics	✓				✓	
DEMQOL		✓	✓*	✓		
EQ-5D-5L		✓	✓	✓	✓	✓
PHQ-9		✓	✓		✓	✓
GAD-7		✓	✓			
GSE		✓	✓			
DFS		✓	✓			
SMAS		✓	✓			
IADL		✓	✓	✓		
HSCRU			✓	✓		
SCQ					✓	✓

For peer review only

Visits to collect outcome data will be arranged with the participant by a researcher, in some cases a participating supporter will assist with these arrangements. All visits will be conducted at a time and location most suitable for the participant. A second visit may be required to complete the outcome measures if the participant becomes tired, which will be organised as soon as possible after the first. Participating supporters may not have the time or capacity to receive a face-to-face visit and follow-up outcome measures may therefore be collected from them over the telephone. Similarly, in the case that a participant is likely to withdraw, a reduced set of outcome measures may be taken by telephone (including DEMQOL and telephone versions of HSCRU and EQ-5D-5L).

Intervention attendance

Records will be kept of all attendances for each participant randomised to the intervention.

Intervention dropout and study withdrawal

If a participant decides to withdraw either from the intervention or the study, this will be recorded. If the participant solely withdrew from the intervention, they will be followed-up unless they explicitly also withdraw consent for follow-up of outcomes (data up to this time will be included in the trial).

Intervention costings

Information on the cost of facilitated group and individual sessions will be collected including hire of local community venues, facilitator salaries and travel, refreshments, and other costs such as administration and materials used.

Fidelity assessment

The fidelity of intervention delivery, as well as of the training and supervision received by facilitators will be assessed according to the intervention protocol and manual. Fidelity checks will adhere to a framework based on that identified by the Behaviour Change Consortium[23] and NICE guidance on behaviour change.[24] The fidelity assessment framework (adapted from Bellg et al, 2004[23]) will assess and monitor for the consistency, facilitator training (in terms of standardised delivery of training and skills acquisition for facilitators), intervention delivery (in terms of standardised delivery between intervention groups) and the receipt of the intervention by intervention group participants.

Training delivery and receipt of the facilitator training will be observed and rated by the same two researchers (the lead for fidelity and one other member of the research team) for inter-rater reliability using a bespoke *Training observation checklist*. To assess facilitator adherence to the manualised intervention and participant receipt of the intervention, a purposive selection of group meetings across sites will also be observed using a *Group observation checklist* based on the contents of the intervention and the training to assess each meeting. Each group selected will be observed on two occasions to identify facilitator drift or changes in participant behavior.

Frequencies will be used to determine the extent to which the training programme received by facilitators maintained fidelity to what was intended. Comparison of intervention delivery between and across sites will also be conducted to check for consistency. Inter-rater reliability between coders will be determined using the Kappa statistic.[44] Similar methods have been used in previous studies, for example the Lifestyle Matters Trial.[45]

Qualitative sub study

An embedded qualitative sub-study will explore the mechanisms of the intervention, for example what elements of the intervention appear to support people to improve their self-management and well-being and what promotes good facilitation of the intervention.

Individual qualitative semi-structured interviews will be conducted with a purposive sample of approximately 20 participants (approximately 10%) from the fidelity intervention groups and with approximately 12 participating supporters, preferably supporters of participants who are also being interviewed. A participant interview schedule and supporter interview schedule will be developed to cover the following themes:

For peer review only - http://bmjopen.bmj.com/site/about/guidelines.xhtml

- Range and nature of issues that influence experiences of taking part in the intervention
- Factors that may mediate or moderate the effectiveness of the intervention
- Perceived skills and competencies required to facilitate the intervention
- The barriers and facilitators to uptake and continued use
- The effect of the intervention upon living with dementia
- The effect of the intervention upon the experiences of supporting someone with dementia

Semi-structured interviews will also be conducted with approximately 20% of all facilitators and supervisors across the sites at the end of their delivery of the intervention. The sample will include a range of sites and facilitators with different levels of experience of delivering the intervention. A facilitator interview schedule and supervisor interview schedule will be developed to cover the following themes:

- What issues promote the effectiveness of intervention facilitation
- The skills and competencies required to facilitate the programme
- The barriers and facilitators to its uptake and continued use
- Factors that may mediate or moderate the effectiveness of the intervention

Researchers undertaking participant and supporter interviews will be trained to use enhanced methods of communication with people with dementia to ensure that meaningful discussion can take place. Transcripts of interviews will undergo respondent validation.

SAMPLE SIZE

The primary outcome for the study is the mean DEMQOL score 8 months post randomisation. Assuming a standard deviation of 11 points for the DEMQOL, a mean difference of 4 or more points is clinically and practically important.[30] The sample size has been calculated to have a 90% power of detecting this 4 point difference (equivalent to a standardised effect size of 0.36) in group mean scores at eight months as being statistically significant at 5% (two sided) level. As the JtD intervention is a facilitator led intervention with a group component, the outcomes of the participants in the same group with the same facilitators may be clustered. With no adjustment for clustering by facilitator the target sample size would be 160 per arm with a total sample size of 320. We have assumed an average cluster size of 8 people with dementia per facilitated group and an intra-cluster correlation of 0.03; this will inflate the sample size by a design effect of 1.21; to 194 per group (388 total sample size) with valid primary outcome data. Assuming at least a 20% loss to follow-up the target sample size for the trial is to randomise to 243 participants in each arm (n=486).

DATA ANALYSIS

Statistical data analysis

As JtD is a pragmatic parallel group randomised trial, with a usual care (control) arm, data will be reported and presented according to a revised CONSORT statement.[46] Statistical analysis will be performed on an intention-to-treat-basis. All exploratory tests will be two-tailed with alpha = 0.05. Baseline demographics and quality of life data will be described and summarised overall and by treatment group.

For peer review only - http://bmjopen.bmj.com/site/about/guidelines.xhtml

The primary analysis will compare mean patient reported DEMQOL scores at 8 months post randomisation between the intervention (JtD) arm and control arms using a mixed effects linear regression model adjusted for DEMQOL baseline score and site and allowing for the clustering of the outcome by the JtD intervention.[47–49] The trial is a partially nested design with comparison of a group therapy (JtD) with individual therapy with clustering in one (intervention) arm. Each person with dementia in the control group (unclustered arm) will be treated as a cluster (singleton) of size one. The cluster indicator will be treated as a random effect. A stratification variable used for randomisation (site) will be included as a fixed factor.[50] A partially clustered mixed effects linear regression model with homoscedastic errors as well as a heteroscedasticity mixed effects linear regression model will also be considered to account for potential differential variability of outcomes between the two treatment groups. A 95% confidence interval (CI) for the mean difference in DEMQOL scores between the intervention and control groups will be calculated together with the associated P-value. A further adjusted analysis may also be performed depending on the observed degree of imbalance in baseline covariates (which are of potential prognostic importance) again using a mixed effects linear regression model. Additional covariates (of potential prognostic importance) include other baseline variables, such as age, gender, PHQ-9, and GAD-7. In the event that there are more than 10 couples (20 participants) from the same household the primary and secondary analyses will be changed to take into account the hierarchical or clustered nature of the data. A multi level mixed effects model will be used; the random effects will be JtD intervention groups (top level) and couple/singles (lower level). Individual participants who are not part of a couple will be treated as clusters of size one.

Participants will be followed up for up to 12 months post randomisation. Mean DEMQOL scores at 12 months follow-up will be compared as described for the primary outcome above.

For the primary outcome, the DEMQOL score at 8 months follow-up, missing data will be imputed through a variety of methods including: regression and multiple imputation as part of a sensitivity analysis.

We will complement the intention to treat (ITT) analysis of the primary outcome with a complier average causal effects analysis (CACE) as a secondary analysis alongside the primary ITT analysis. Compliance will be defined as a binary variable with participants who attend at least 10 of the 16 JtD sessions (both individual and group sessions combined) regarded as being compliant.

There are no planned interim statistical analyses or formal stopping rules in relation to efficacy.

Secondary outcome measures

Secondary outcomes at 8 and 12 months post-randomisation will be compared between the intervention and control groups using a mixed effects linear regression model as for the primary outcome. A 95% CI for the mean difference in this parameter between the treatment groups will also be calculated together with the associated P-value.

Outcome measures for the participating supporters at 8 months will be compared between the intervention and control groups using a mixed effects linear regression model. The mean difference in outcome with associated 95% CI and P-value will be presented for: a) the baseline (specific to the

For peer review only - http://bmjopen.bmj.com/site/about/guidelines.xhtml

secondary outcome) and site adjusted analysis and b) adjusted analysis with additional covariates in addition to a).

Subgroup analysis

A subgroup analysis using a mixed effect linear regression model, with the primary outcome (DEMQOL) at 8 months post randomisation as the response will be carried out. We will use an interaction statistical test between the randomised intervention group and subgroup to directly examine the strength of evidence for the treatment difference between the treatment groups varying between subgroups. Supporter involvement (yes or no) will be the only a priori defined sub groups to be considered for interaction test.

Qualitative analysis

Framework analysis[51] will be applied to all interviews.[52] For the purposes of reporting, confidentiality will be maintained by using unique participant identifiers and removing identifiable information. A thematic framework will be agreed by two researchers and an index developed for transcript coding. This will follow the five stages of framework analysis.[51] Findings will be used to identify emergent factors that influence the uptake and impact of the intervention as well as explore potential explanations for the quantitative findings.[52] Further analysis will also be undertaken to triangulate the qualitative data (between facilitator/supervisor and participant/supporter interviews) as well as the fidelity and qualitative data in order to look for between source similarities and divergences. Analysis workshops will be held with persons living with dementia and their supporters to respond to and help validate the qualitative analysis. The workshop outcomes will be used to refine the qualitative analysis.

Health economics evaluation

A trial based economic evaluation will be undertaken of an intention-to-treat comparison of the costs and outcomes of the two trial arms. A cost-effectiveness analysis will be undertaken of the incremental cost per Quality Adjusted Life Year (QALYs) of the JtD intervention compared with usual care provided through NHS memory services. QALYs will be calculated using the EQ-5D-5L preference-based index administered at baseline, 8 and 12 months. A sensitivity analysis will be undertaken using utility values from the DEMQOL-U, which can be derived from responses to the DEMQOL questionnaire.[30] The total cost of the intervention will be estimated at the individual participant level and will include the costs of providing the intervention and the subsequent consequences for the use of routine health and social care services. The average cost per attendance will be calculated and this estimate will be applied to the actual number of group and individual sessions that each participant attended.

The use of services by trial participants will be collected in detail using a Health and Social Care Resource Use (HSCRU) questionnaire administered at 8 and 12 months post randomisation. Service use will be costed using the most recent National Reference Cost Data and Unit Costs of Health and Social Care.[53,54] Missing data will be dealt with using multiple imputation for EQ-5D-5L, DEMQOL-U and resource use data.[55] A random effects linear regression model, accounting for clustering, will be fitted, the model will include baseline scores for EQ-5D-5L and baseline costs. The central

For peer review only - http://bmjopen.bmj.com/site/about/guidelines.xhtml

analysis of mean incremental costs per QALY will be subjected to a full sensitivity analysis of key parameters including the measure used to estimate QALYs and number of participants at the weekly sessions. A full probabilistic sensitivity analysis will be performed to examine the probability of cost-effectiveness of the intervention for the NHS for different levels of costs and QALY gains.[56]

DATA MANAGEMENT, CONFIDENTIALITY AND SHARING

Sheffield Clinical Trials Research Unit (CTRU) will provide data management services. Data will be entered remotely on to a centralised web-based data capture system (Prospect) by university researchers and authorised staff at participating NHS sites. The Case Report Form captures trial data and has been specifically designed for this trial. Access to Prospect is controlled by usernames and encrypted passwords. Prospect provides a full electronic audit trail, as well as validation features which will be used to monitor study data quality. The identity of participants will be protected by the removal of any identifiable data prior to dissemination of information, and no identifiable data will be transferred to the statistician or health economist. All participating NHS Sites will be subject to data monitoring reviews to check data entry, consent and eligibility, amongst other items.

Data sharing

JtD trial data will be held and available for five years after the end of the trial (November 2019). JtD Trial Data will not be archived in a repository, instead data will be released on a case-by-case basis. We shall make data available to the scientific community with as few restrictions as feasible. Data access requests will be reviewed and authorised by a sub-committee of the Trial Management Group (TMG) during the trial and by the Sheffield CTRU after the trial has ended. Access requests will be considered against pre-determined criteria and data sharing will only take place if this aligns with the consent provided by JtD participants. Data will be anonymised prior to being shared.

ETHICS, GOVERNANCE AND SAFETY

Ethical issues

There are two key ethical issues to take into account. The first is the potential need to break confidentiality where there is a risk of harm. We will request consent to contact the participants or supporter's GP or other health professional in situations where researchers are concerned that there might be risk of harm. For example, two outcome measures used on the study may indicate a need to treat anxiety (GAD-7) or depression (PHQ-9) clinically. Additionally, concerns regarding participant safety may also be raised at any stage of the study; e.g. observed deterioration in mental or physical state of participants, safeguarding issues, or of a risk to self or others. We will work alongside local Trust procedures to report any risks appropriately. Local site investigators with clinical backgrounds will be asked to provide advice when appropriate. The responsible healthcare professional or PIs will be able to recommend the withdrawal of the participant if they feel it is appropriate. We will record all actions taken.

The second is the risk that during the trial, people with dementia may lose the capacity to consent to continuing participation. As part of the consent process, people living with dementia will be asked to nominate a person to act as a consultee. If the person with dementia loses capacity during the trial, the consultee will be contacted to give their independent assessment of whether the person has the

For peer review only - http://bmjopen.bmj.com/site/about/guidelines.xhtml

capacity to continue or not with the study. We will record information about the activation of the consultee pathway on the trial.

Governance

The trial is coordinated by the Sheffield CTRU on behalf of the Sponsor. The sponsor of the trial is Sheffield Health and Social Care Foundation Trust, Fulwood House, Old Fulwood Road, Sheffield, S10 3TH. The JtD TMG contains project co-applicants, members of the data management team, the Sponsor, Trial Manager and other representatives, and oversees the operation of the trial and enables communication throughout the Trial, for example to disseminate protocol amendments. An independent Trial Steering Committee (TSC), comprised of an independent statistician, PPI representative and a Senior Clinical Research Associate, provides overall supervision of the trial, advises the CI, oversees protocol modifications, monitors the trial's progress and if necessary closes the trial. An independent Data Monitoring and Ethics Committee (DMEC) comprised of two independent statisticians and an Occupational Therapist Clinical Researcher, reviews the trial protocol, monitors patient safety and advises the TSC if they feel the trial should be prematurely closed.

Safety

A Serious Adverse Event (SAE) reporting system will be used on the Trial. Adverse Events are not anticipated as a consequence of the intervention and will not be monitored. An SAE either:

a) results in death,
b) is life-threatening (subject at immediate risk of death),
c) requires hospitalisation or prolongation of existing hospitalisation,
d) results in persistent or significant disability or incapacity, or
e) is otherwise considered medically significant by the investigator.

All SAEs will be assessed to see if they are related to the intervention or other trial procedures, and if they are the Sponsor and Research Ethics Committee will be immediately informed. SAEs are periodically reported to the trial's DMEC.

Additionally, we consider safety of the researchers to be extremely important and have developed a lone worker policy.

DISSEMINATION

The results of this study will be communicated in relevant academic and professional journals, conferences and workshops, and via websites and social media, ensuring reach to all stakeholders (people living with the condition, professionals, commissioners and academics). A short film will be created to illustrate experiences of participation from the perspectives of people living with the condition, and those involved in delivery of the intervention. The manualised intervention will be refined and made available on the website in an open access format.

AUTHOR CONTRIBUTIONS

For peer review only - http://bmjopen.bmj.com/site/about/guidelines.xhtml

AF, GM, CCo, KS, EL, SW, TY, CCr, EMC, AL, TD and KB co-wrote the original Trial Protocol. JW led the development of the Protocol for publication. JBD and KS developed the sections on fidelity and qualitative analysis. JBD developed the section on PPI. BDT adapted the introductory section, BJT the methods section, EY the outcomes section and EL the statistical analysis section. All authors contributed to reviewing and revising the draft versions prior to submission.

ACKNOWLEDGEMENTS

We would like to thank the following people for assisting with the original project application: Martin Orrell, Institute of Mental Health, The University of Nottingham; Daniel Blackburn, Sheffield Institute for Translational Neuroscience, The University of Sheffield; and Diana Papaioannou, Sheffield Clinical Trials Research Unit, School of Health and Related Research, The University of Sheffield.

Additionally, we would like to thank Peter Bowie, Old Age Psychiatry, Sheffield Health and Social Care NHS Foundation Trust, Sheffield, for advising on clinical aspects; and Nicholas Bell, Sheffield Health and Social Care NHS Foundation Trust, for acting on behalf of the sponsor and advising on trial procedures.

FUNDING STATEMENT

This study was funded by the NIHR Health Technology Assessment Programme (project number 14/140/80). The views expressed are those of the author(s) and not necessarily those of the NHS, the NIHR or the Department of Health.

COMPETING INTERESTS

All authors, with the exception of those below, declare no competing interests.

- SW, TY, CCo, JW, AF, AL, EL, BJT, BDT, EY as ScHARR has contracts and/or research grants with the Department of Health, NIHR, MRC and NICE.
- SW, CCo and TY are co-applicants or co-investigators on NIHR portfolio grants (NIHR Research Design Service for Yorkshire and Humber; HTA, RfPB, PHR; SDO) and grants from the MRC.
- SW and TY also receive external examining fees from various UK higher education institutes.
- SW receives book royalties from publishers including John Wiley and Sons Ltd and Blackwell Publishing.
- BDT is a Consultant for Arch research in the area of dementia.

ETHICS APPROVAL

This study was approved by Leeds East Research Ethics Committee, on 01/07/16, reference number 16/YH/0238. Health Research Authority approval was provided for the study to commence on 25/08/16. The current Protocol version is v7, 5th December 2018.

REFERENCES

1 World Health Organisation. The global burden of disease: 2004 update. Geneva: World Health Organisation 2008.

For peer review only

2 Knapp M, Comas-Herrera A, Somani A, et al. Dementia: international comparisons – summary report to the National Audit Office. London: London School of Economics 2007.

3 Department of Health. Prime Minister's challenge on dementia 2020. London: Department of Health 2015. https://www.gov.uk/government/uploads/system/uploads/attachment_data/file/406076/Dementia_vision.pdf (accessed 26 Sep 2018).

4 Department of Health. Living well with dementia : A National Dementia Strategy. London: Department of Health 2009. https://www.gov.uk/government/publications/living-well-with-dementia-a-national-dementia-strategy (accessed 26 Sep 2018).

5 Hodge AS, Hailey E. English National Memory Clinics Audit Report. London: Royal College of Psychiatrists 2013. http://www.rcpsych.ac.uk/pdf/English National Memory Clinics Audit Report 2013.pdf (accessed 30 Oct 2018).

6 Banerjee S, Wittenberg R. Clinical and cost effectiveness of services for early diagnosis and intervention in dementia. *Int J Geriatr Psychiatry* 2009;24:748–54. doi:10.1002/gps.2191

7 Copland E, Hodge S, Clary L, *et al.* Memory Services National Accreditation Programme (MSNAP): Standards for memory services. London: Royal College of Psychiatrists 2018. https://www.rcpsych.ac.uk/pdf/MSNAP_Standards_6_Edition_2018.pdf (accessed 16 Oct 2018).

8 NHS England. Well Pathway for Dementia. Leeds: NHS England 2017. https://www.england.nhs.uk/mentalhealth/wp-content/uploads/sites/29/2016/03/dementia-well-pathway.pdf (accessed 30 Oct 2018).

9 National Institute for Health and Clinical Excellence (NIHCE). Donepezil, galantamine, rivastigmine, and memantine for the treatment of Alzheimer's Disease. London: NIHCE 2011. http://www.nice.org.uk/guidance/ta217/resources/guidance-donepezilgalantaminerivastigmineandmemantine-for-the-treatment-of-alzheimers-disease-pdf (accessed 26 Sep 2018).

10 Woods B, Aguirre E, Spector AE, et al. Cognitive stimulation to improve cognitive functioning in people with dementia. *Cochrane Database Syst Rev* 2012;15. doi:10.1002/14651858.CD005562.pub2

11 Moniz-Cook E, Vernooij-Dassen M, Woods B, et al. Psychosocial interventions in dementia care research: The INTERDEM manifesto. *Aging Ment Health* 2011;15:283–90. doi:10.1080/13607863.2010.543665

12 McDermott O, Charlesworth G, Hogervorst E, et al. Psychosocial interventions for people with dementia: a synthesis of systematic reviews. *Aging Ment Health* Published Online First:17 January 2018. doi:10.1080/13607863.2017.1423031

13 Clare L, Woods RT. Cognitive training and cognitive rehabilitation for people with early-stage Alzheimer's disease: A review. *Neuropsychol Rehabil* 2004;14:385–401. doi:10.1080/09602010443000074

14 Graff MJL, Vernooij-Dassen MJM, Thijssen M, et al. Community based occupational therapy for patients with dementia and their care givers: randomised controlled trial. *BMJ* 2006;333:1196. doi:10.1136/bmj.39001.688843.BE

15 Vernooij-Dassen M, Olde Rikkert MG. Personal disease management in dementia care. *Int J*

For peer review only - http://bmjopen.bmj.com/site/about/guidelines.xhtml

Geriatr Psychiatry 2004;19:715–7. doi:10.1002/gps.1149

16 Moniz-Cook E, Manthorpe J, eds. Early Psychosocial Interventions in Dementia. London: Jessica Kingsley Publishers 2008.

17 Quinn C, Toms G, Anderson D, et al. A review of self-management interventions for people with dementia and mild cognitive impairment. *J Appl Gerontol* 2016;35:1154–88. doi:10.1177/0733464814566852

18 Quinn C, Toms G, Jones C, et al. A pilot randomized controlled trial of a self-management group intervention for people with early-stage dementia (The SMART study). *Int Psychogeriatr* 2016;28:787–800. doi:10.1017/S1041610215002094

19 Martin F, Turner A, Wallace LM, et al. Qualitative evaluation of a self-management intervention for people in the early stage of dementia. *Dementia* 2015;14:418–35. doi:10.1177/1471301213498387

20 Clarke CL, Keyes SE, Wilkinson H, et al. Healthbridge: The National Evaluation of Peer Support Networks and Dementia Advisers in Implementation of the National Dementia Strategy for England. London: Department of Health 2013.

21 Chakkalackal L, Kalathil J. Evaluation report: Peer support groups to facilitate self-help coping strategies for people with dementia in extra care housing. London: Mental Health Foundation 2014.

22 Sprange K, Mountain GA, Shortland K, et al. Journeying through Dementia, a community-based self-management intervention for people aged 65 years and over: a feasibility study to inform a future trial. *Pilot Feasibility Stud* 2015;1:42. doi:10.1186/s40814-015-0039-6

23 Bellg AJ, Resnick B, Minicucci DS, et al. Enhancing treatment fidelity in health behavior change studies: Best practices and recommendations from the NIH Behavior Change Consortium. *Health Psychology* 2004;23:443–51. doi:10.1037/0278-6133.23.5.443

24 National Institute for Health and Care Excellence (NIHCE). Behaviour Change at population, community and individual levels. Public Health Guidance 6. London: NIHCE 2007.

25 Craig P, Dieppe P, Macintyre S, et al. Developing and evaluating complex interventions: new guidance. London: Medical Research Council 2008. http://www.mrc.ac.uk/Utilities/Documentrecord/index.htm?d=MRC004871 (Accessed 28 Sep 2018).

26 ISRCTN Registry. ISRCTN17993825 Journeying through dementia: exploring the clinical and cost-effectiveness of a group self-management intervention for people in the early stages of dementia. 2019. http://www.isrctn.com/ISRCTN17993825 (accessed 15 Jan 2019).

27 Alzheimer's Disease International. Participation in dementia trials and studies: Challenges and recommendations. 2014. https://www.alz.co.uk/sites/default/files/pdfs/dementia-trials.pdf (accessed 28 Sept 2018).

28 Herschell AD, Kolko DJ, Baumann BL, et al. The role of therapist training in the implementation of psychosocial treatments: A review and critique with recommendations. *Clin Psychol Rev* 2010;30:448–66. doi:10.1016/j.cpr.2010.02.005

29 Mountain GA, Craig CL. What should be in a self-management programme for people with early dementia? *Aging Ment Health* 2012;16:576–83. doi:10.1080/13607863.2011.651430

For peer review only

30 Mulhern B, Rowen D, Brazier J, et al. Development of DEMQOL-U and DEMQOL-PROXY-U: Generation of preference-based indices from DEMQOL and DEMQOL-PROXY for use in economic evaluation. *Health Technol Assess (Rockv)* 2013;17:1–140. doi:10.3310/hta17050

31 Smith S, Lamping D, Banerjee S, et al. Measurement of health-related quality of life for people with dementia: development of a new instrument (DEMQOL) and an evaluation of current methodology. *Health Technol Assess (Rockv)* 2005;9:1–93. doi:https://doi.org/10.3310/hta9100

32 Herdman M, Gudex C, Lloyd A, et al. Development and preliminary testing of the new five-level version of EQ-5D (EQ-5D-5L). *Qual Life Res* 2011;20:1727–36. doi:10.1007/s11136-011-9903-x

33 Hounsome N, Orrell M, Edwards RT. EQ-5D as a quality of life measure in people with dementia and their carers: Evidence and key issues. *Value Health* 2011;14:390–9. doi:10.1016/j.jval.2010.08.002

34 Spitzer RL, Williams JBW, Kroenke K. Test Review: Patient Health Questionnaire-9 (PHQ-9). *Rehabil Couns Bull* 2014;57:246–8. doi:10.1177/0034355213515305

35 Kroenke K, Spitzer RL, Williams JBW. The PHQ-9: Validity of a Brief Depression Severity Measure. *J Gen Intern Med* 2001;16:606–13. doi:10.1046/j.1525-1497.2001.016009606.x

36 Löwe B, Decker O, Müller S, et al. Validation and standardization of the generalized anxiety disorder screener (GAD-7) in the general population. *Medical Care* 2008;46:266–74. doi:10.1097/MLR.0b013e318160d093

37 Schwarzer R, Jerusalem M. Generalized Self-Efficacy scale. In: Weinman J, Wright S, Johnston M, eds. Measures in health psychology: A user's portfolio. Causal and control beliefs. Windsor: NFER-NELSON 1995: 35–7.

38 Diener E, Wirtz D, Tov W, et al. New measures of well-being: Flourishing and positive and negative feelings. In: Diener E, ed. Social Indicators Research Series. New York: Springer Science + Business Media 2009: 247–66.

39 Schuurmans H, Steverink N, Frieswijk N, et al. How to Measure Self-Management Abilities in Older People by Self-report. The Development of the SMAS-30. *Qual Life Res* 2005;14(10):2215–2228. Doi: doi:10.1007/s11136-005-8166-9

40 Lawton MP, Brody EM. Assessment of Older People : Self-Maintaining and Instrumental Activities of Daily Living 1. *Gerontologist* 1969;9:179–86. doi:10.1093/geront/9.3_Part_1.179

41 Vernooij-Dassen MJFJ, Persoon JMG, Felling AJA. Predictors of sense of competence in caregivers of demented persons. *Soc Sci Med* 1996;43:41–9. doi:10.1016/0277-9536(95)00332-0

42 Jansen APD, van Hout HPJ, van Marwijk HWJ, et al. Sense of competence questionnaire among informal caregivers of older adults with dementia symptoms: A psychometric evaluation. *Clin Pract Epidemiol Ment Health* 2007;3:1–11. doi:10.1186/1745-0179-3-11

43 Moniz-Cook E, Vernooij-Dassen M, Woods R, et al. A European consensus on outcome measures for psychosocial intervention research in dementia care. *Aging Ment Heal* 2008;1:14–29. doi:10.1080/13607860801919850

44 McHugh ML. Interrater reliability: the kappa statistic. *Biochem Med (Zagreb)* 2012;22:276–82.

For peer review only

doi:10.11613/BM.2012.031

45 Sprange K., Mountain GA, Brazier J, et al. Lifestyle Matters for maintenance of health and wellbeing in people aged 65 years and over: study protocol for a randomised controlled trial. *Trials* 2013;14:302. doi:10.1186/1745-6215-14-302

46 Schulz KF, Altman DG, Moher D. CONSORT 2010 Statement : updated guidelines for reporting parallel group randomised trials. *BMJ* 2010;340:332. doi:10.1136/bmj.c332

47 Baldwin SA, Bauer DJ, Stice E, et al. Evaluating models for partially clustered designs. *Psychol Methods* 2011;16:149–65. doi:10.1037/a0023464

48 Walters SJ. Therapist effects in randomised controlled trials: What to do about them. *J Clin Nurs* 2010;19:1102–12. doi:10.1111/j.1365-2702.2009.03067.x

49 Roberts C, Batistatou E, Roberts SA. Design and analysis of trials with a partially nested design and a binary outcome measure. *Stat Med* 2015;35:1616–36. doi:10.1002/sim.6828

50 Kahan BC, Morris TP. Improper analysis of trials randomised using stratified blocks or minimisation. *Stat Med* 2012;31:328–40. doi:10.1002/sim.4431

51 Ritchie J, Spencer L. Qualitative Data Analysis for Applied Policy Research. In: Humberman AM, Miles MB, eds. The Qualitative Researcher's Companion. London: SAGE Publications 2002: 305–29. doi:10.4135/9781412986274

52 Cooper C, Ketley D, Livingston G. Systematic review and meta-analysis to estimate potential recruitment to dementia intervention studies. *Int J Geriatr Psychiatry* 2014;29:515–25. doi:10.1002/gps.4034

53 NHS Improvement. Reference Costs 2017/18. 2018. https://improvement.nhs.uk/resources/reference-costs/ (accessed 15 Jan 2019).

54 Curtis L, Burns A. Unit Costs of Health & Social Care 2018. Canterbury: University of Kent 2018.

55 Schafer JL. Multiple imputation: A primer. *Stat Methods Med Res* 1999;8:3–15. doi:10.1191/096228099671525676

56 Van Hout BA, Al MJ, Gordon GS, et al. Costs, effects and C/E-ratios alongside a clinical trial. *Health Econ* 1994;3:309–19. doi:10.1002/hec.4730030505

FIGURE LEGENDS

Figure 1: Participant flow through the study

For peer review only - http://bmjopen.bmj.com/site/about/guidelines.xhtml

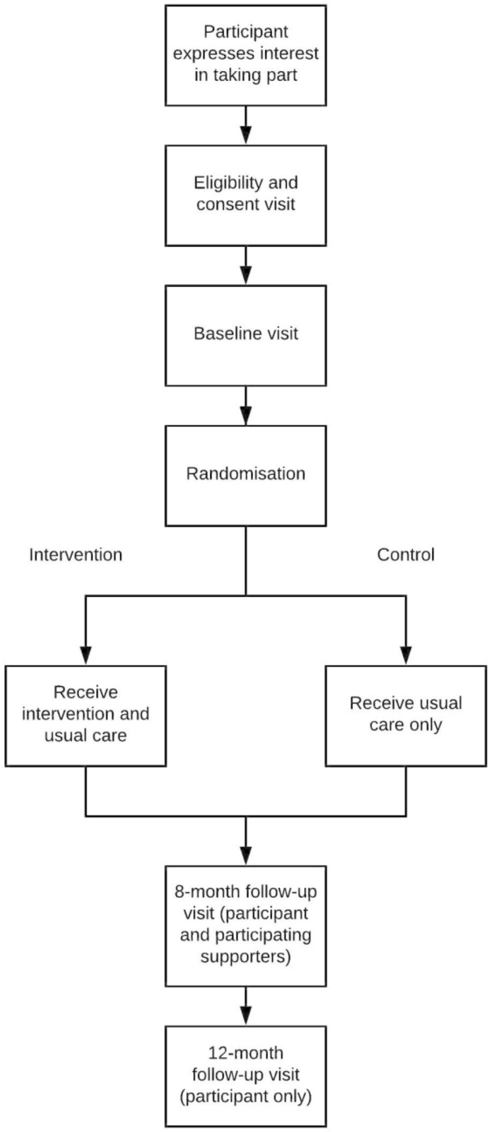

Figure 1 Participant flow through the study

92x190mm (300 x 300 DPI)

SPIRIT

STANDARD PROTOCOL ITEMS: RECOMMENDATIONS FOR INTERVENTIONAL TRIALS

SPIRIT 2013 Checklist: Recommended items to address in a clinical trial protocol and related documents*

Section/item	Item No	Description	Addressed on page number
**Administrative information**			
Title	1	Descriptive title identifying the study design, population, interventions, and, if applicable, trial acronym	_________i____
Trial registration	2a	Trial identifier and registry name. If not yet registered, name of intended registry	_________2_____
	2b	All items from the World Health Organization Trial Registration Data Set	____See 2a_____
Protocol version	3	Date and version identifier	________14______
Funding	4	Sources and types of financial, material, and other support	_____14______
Roles and responsibilities	5a	Names, affiliations, and roles of protocol contributors	______i-ii_______
	5b	Name and contact information for the trial sponsor	____13_______
	5c	Role of study sponsor and funders, if any, in study design; collection, management, analysis, and interpretation of data; writing of the report; and the decision to submit the report for publication, including whether they will have ultimate authority over any of these activities	_____n/a_______
	5d	Composition, roles, and responsibilities of the coordinating centre, steering committee, endpoint adjudication committee, data management team, and other individuals or groups overseeing the trial, if applicable (see Item 21a for data monitoring committee)	_____13________

For peer review only

**Introduction**			
Background and rationale	6a	Description of research question and justification for undertaking the trial, including summary of relevant studies (published and unpublished) examining benefits and harms for each intervention	____2-3________
	6b	Explanation for choice of comparators	____2-3________
Objectives	7	Specific objectives or hypotheses	____3_________
Trial design	8	Description of trial design including type of trial (eg, parallel group, crossover, factorial, single group), allocation ratio, and framework (eg, superiority, equivalence, noninferiority, exploratory)	____3-4________
**Methods: Participants, interventions, and outcomes**			
Study setting	9	Description of study settings (eg, community clinic, academic hospital) and list of countries where data will be collected. Reference to where list of study sites can be obtained	______5_______
Eligibility criteria	10	Inclusion and exclusion criteria for participants. If applicable, eligibility criteria for study centres and individuals who will perform the interventions (eg, surgeons, psychotherapists)	____4-5________
Interventions	11a	Interventions for each group with sufficient detail to allow replication, including how and when they will be administered	____5-6____
	11b	Criteria for discontinuing or modifying allocated interventions for a given trial participant (eg, drug dose change in response to harms, participant request, or improving/worsening disease)	_______n/a_____
	11c	Strategies to improve adherence to intervention protocols, and any procedures for monitoring adherence (eg, drug tablet return, laboratory tests)	______8-9_____
	11d	Relevant concomitant care and interventions that are permitted or prohibited during the trial	______6_____
Outcomes	12	Primary, secondary, and other outcomes, including the specific measurement variable (eg, systolic blood pressure), analysis metric (eg, change from baseline, final value, time to event), method of aggregation (eg, median, proportion), and time point for each outcome. Explanation of the clinical relevance of chosen efficacy and harm outcomes is strongly recommended	_____6-7__
Participant timeline	13	Time schedule of enrolment, interventions (including any run-ins and washouts), assessments, and visits for participants. A schematic diagram is highly recommended (see Figure)	____Table 1 and figure 1_____

For peer review only - http://bmjopen.bmj.com/site/about/guidelines.xhtml

Sample size	14	Estimated number of participants needed to achieve study objectives and how it was determined, including clinical and statistical assumptions supporting any sample size calculations	___9______
Recruitment	15	Strategies for achieving adequate participant enrolment to reach target sample size	_____5_______

Methods: Assignment of interventions (for controlled trials)

Allocation:

Sequence generation	16a	Method of generating the allocation sequence (eg, computer-generated random numbers), and list of any factors for stratification. To reduce predictability of a random sequence, details of any planned restriction (eg, blocking) should be provided in a separate document that is unavailable to those who enrol participants or assign interventions	_____4________
Allocation concealment mechanism	16b	Mechanism of implementing the allocation sequence (eg, central telephone; sequentially numbered, opaque, sealed envelopes), describing any steps to conceal the sequence until interventions are assigned	____4_________
Implementation	16c	Who will generate the allocation sequence, who will enrol participants, and who will assign participants to interventions	____4_______
Blinding (masking)	17a	Who will be blinded after assignment to interventions (eg, trial participants, care providers, outcome assessors, data analysts), and how	___4______
	17b	If blinded, circumstances under which unblinding is permissible, and procedure for revealing a participant's allocated intervention during the trial	______4_______

Methods: Data collection, management, and analysis

Data collection methods	18a	Plans for assessment and collection of outcome, baseline, and other trial data, including any related processes to promote data quality (eg, duplicate measurements, training of assessors) and a description of study instruments (eg, questionnaires, laboratory tests) along with their reliability and validity, if known. Reference to where data collection forms can be found, if not in the protocol	____6-8_______
	18b	Plans to promote participant retention and complete follow-up, including list of any outcome data to be collected for participants who discontinue or deviate from intervention protocols	____6-7_______

Data management	19	Plans for data entry, coding, security, and storage, including any related processes to promote data quality (eg, double data entry; range checks for data values). Reference to where details of data management procedures can be found, if not in the protocol	__12
Statistical methods	20a	Statistical methods for analysing primary and secondary outcomes. Reference to where other details of the statistical analysis plan can be found, if not in the protocol	____9-11_
	20b	Methods for any additional analyses (eg, subgroup and adjusted analyses)	__11_
	20c	Definition of analysis population relating to protocol non-adherence (eg, as randomised analysis), and any statistical methods to handle missing data (eg, multiple imputation)	_10-11___
**Methods: Monitoring**			
Data monitoring	21a	Composition of data monitoring committee (DMC); summary of its role and reporting structure; statement of whether it is independent from the sponsor and competing interests; and reference to where further details about its charter can be found, if not in the protocol. Alternatively, an explanation of why a DMC is not needed	____13_____
	21b	Description of any interim analyses and stopping guidelines, including who will have access to these interim results and make the final decision to terminate the trial	______n/a______
Harms	22	Plans for collecting, assessing, reporting, and managing solicited and spontaneously reported adverse events and other unintended effects of trial interventions or trial conduct	___12-13____
Auditing	23	Frequency and procedures for auditing trial conduct, if any, and whether the process will be independent from investigators and the sponsor	____12______
**Ethics and dissemination**			
Research ethics approval	24	Plans for seeking research ethics committee/institutional review board (REC/IRB) approval	______14_______
Protocol amendments	25	Plans for communicating important protocol modifications (eg, changes to eligibility criteria, outcomes, analyses) to relevant parties (eg, investigators, REC/IRBs, trial participants, trial registries, journals, regulators)	_______13______

For peer review only - http://bmjopen.bmj.com/site/about/guidelines.xhtml

Consent or assent	26a	Who will obtain informed consent or assent from potential trial participants or authorised surrogates, and how (see Item 32)	______5______
	26b	Additional consent provisions for collection and use of participant data and biological specimens in ancillary studies, if applicable	__See supplementary information__
Confidentiality	27	How personal information about potential and enrolled participants will be collected, shared, and maintained in order to protect confidentiality before, during, and after the trial	___12-13_____
Declaration of interests	28	Financial and other competing interests for principal investigators for the overall trial and each study site	_____14______
Access to data	29	Statement of who will have access to the final trial dataset, and disclosure of contractual agreements that limit such access for investigators	_____12____
Ancillary and post-trial care	30	Provisions, if any, for ancillary and post-trial care, and for compensation to those who suffer harm from trial participation	_____n/a______
Dissemination policy	31a	Plans for investigators and sponsor to communicate trial results to participants, healthcare professionals, the public, and other relevant groups (eg, via publication, reporting in results databases, or other data sharing arrangements), including any publication restrictions	_____13___
	31b	Authorship eligibility guidelines and any intended use of professional writers	______n/a______
	31c	Plans, if any, for granting public access to the full protocol, participant-level dataset, and statistical code	______12______
**Appendices**			
Informed consent materials	32	Model consent form and other related documentation given to participants and authorised surrogates	See supplementary materials_____
Biological specimens	33	Plans for collection, laboratory evaluation, and storage of biological specimens for genetic or molecular analysis in the current trial and for future use in ancillary studies, if applicable	_____n/a_______ _

For peer review only - http://bmjopen.bmj.com/site/about/guidelines.xhtml

*It is strongly recommended that this checklist be read in conjunction with the SPIRIT 2013 Explanation & Elaboration for important clarification on the items. Amendments to the protocol should be tracked and dated. The SPIRIT checklist is copyrighted by the SPIRIT Group under the Creative Commons "Attribution-NonCommercial-NoDerivs 3.0 Unported" license.

For peer review only

BMJ Open

A study protocol for a randomised controlled trial of the clinical and cost-effectiveness of the Journeying through Dementia (JtD) intervention compared to usual care

Journal:	*BMJ Open*
Manuscript ID	bmjopen-2019-029207.R1
Article Type:	Protocol
Date Submitted by the Author:	25-Jun-2019
Complete List of Authors:	Wright, Jessica; University of Sheffield, Clinical Trials Research Unit<br>Foster, Alexis; University of Sheffield, School of Health and Related Research<br>Cooper, Cindy; The University of Sheffield, Sheffield Clinical Trials Research Unit, School of Health and Related Research<br>Sprange, Kirsty; The University of Nottingham, Nottingham Clinical Trials Research Unit<br>Walters, Stephen; University of Sheffield, ScHARR<br>Berry, Katherine; The University of Manchester, School of Psychological Sciences<br>Moniz-Cook , Esme ; The University of Hull, School of Health and Social Work<br>Loban, Amanda; University of Sheffield, School of Health and Related Research<br>Young, Tracey; The University of Sheffield, School of Health and Related Research<br>Craig, Claire; Sheffield Hallam University<br>Dening, Tom; University of Nottingham, Division of Psychiatry & Applied Psychology, School of Medicine<br>Lee, Ellen; University of Sheffield, Clinical Trials Research Unit<br>Beresford-Dent, Julie; University of Bradford, Centre for Applied Dementia Studies<br>Thompson, Benjamin; The University of Sheffield, School of Health and Related Research<br>Young, Emma; The University of Sheffield, School of Health and Related Research<br>Thomas, Benjamin; The University of Sheffield, School of Health and Related Research<br>Mountain, Gail; University of Bradford, Centre for Applied Dementia Studies, Faculty of Health Studies
**Primary Subject Heading**:	Geriatric medicine
Secondary Subject Heading:	Mental health, Health economics, Qualitative research
Keywords:	Randomised Controlled Trial, Dementia < NEUROLOGY, Research Protocol, Post diagnostic support, Self-management, Well-being

For peer review only - http://bmjopen.bmj.com/site/about/guidelines.xhtml

TITLE PAGE

Title

A study protocol for a randomised controlled trial of the clinical and cost-effectiveness of the Journeying through Dementia (JtD) intervention compared to usual care

Corresponding author

Jessica Wright
Journeying Through Dementia (JtD) Trial,
Sheffield Clinical Trials Research Unit,
ScHARR, The University of Sheffield,
Regent's Court,
30 Regent St, Sheffield S1 4DA
Telephone: 0114 222 4304
Email: Jessica.wright@sheffield.ac.uk

Author Names/Affiliations

Jessica Wright, Sheffield Clinical Trials Research Unit, School of Health and Related Research, The University of Sheffield, Sheffield, UK

Alexis Foster, School of Health and Related Research, The University of Sheffield, Sheffield, UK

Cindy Cooper, Sheffield Clinical Trials Research Unit, School of Health and Related Research, The University of Sheffield, Sheffield, UK

Kirsty Sprange, Nottingham Clinical Trials Research Unit, The University of Nottingham, Nottingham, UK

Stephen Walters, School of Health and Related Research, The University of Sheffield, Sheffield, UK

Katherine Berry, Manchester Institute for Collaborative Research on Ageing, The University of Manchester, Manchester, UK

Esme Moniz-Cook, School of Health and Social Work, The University of Hull, Hull, UK

Amanda Loban, Sheffield Clinical Trials Research Unit, School of Health and Related Research, The University of Sheffield, Sheffield, UK

Tracey Young, School of Health and Related Research, The University of Sheffield, Sheffield, UK

Claire Craig, Art & Design Research Centre, Sheffield Hallam University, Sheffield, UK

Tom Dening, Division of Psychiatry & Applied Psychology, School of Medicine, University of Nottingham, Nottingham, UK

Ellen Lee, Sheffield Clinical Trials Research Unit, School of Health and Related Research, The University of Sheffield, Sheffield, UK

For peer review only - http://bmjopen.bmj.com/site/about/guidelines.xhtml

Julie Beresford-Dent, Centre for Applied Dementia Studies, University of Bradford, Bradford, UK

Benjamin John Thompson, Sheffield Clinical Trials Research Unit, School of Health and Related Research, The University of Sheffield, Sheffield, UK

Emma Louise Young, Sheffield Clinical Trials Research Unit, School of Health and Related Research, The University of Sheffield, Sheffield, UK

Benjamin David Thomas, Sheffield Clinical Trials Research Unit, School of Health and Related Research, The University of Sheffield, Sheffield, UK

Gail Mountain, Centre for Applied Dementia Studies, Faculty of Health Studies, University of Bradford, Bradford, UK

For peer review only

Word Count: 6589 (excluding references, abstract, tables and figures)

For peer review only - http://bmjopen.bmj.com/site/about/guidelines.xhtml

ABSTRACT

Introduction

Services are being encouraged to provide post diagnostic treatment to those with dementia but the availability of evidence-based interventions following diagnosis has not kept pace with increase in demand. To address this need, the Journeying through Dementia (JtD) intervention was created. A randomised controlled trial (RCT), based on a pilot study, is in progress.

Methods and analysis

The RCT is a pragmatic, two-arm, parallel group trial designed to test the clinical and cost-effectiveness of JtD compared to usual care. Recruitment will be through NHS services, third sector organisations and Join Dementia Research. The sample size is 486 randomised (243 to usual care and 243 to the intervention usual care). Participants can choose to ask a friend or relative (supporter) to become involved in the study. The primary outcome measure for participants is Dementia Related Quality of Life (DEMQOL), collected at baseline and at 8 months' post randomisation. Secondary outcome measures will be collected from participants and supporters at those visits. Participants will also be followed-up at 12 months' post randomisation with a reduced set of measures. A process evaluation will be conducted through qualitative and fidelity sub-studies.

Analyses will compare the two arms of the trial on an intention to treat as allocated basis. The primary analyses will compare the mean DEMQOL scores of the participants at 8 months between the two study arms. A cost-effectiveness analysis will consider the incremental cost per Quality Adjusted Life Years of the intervention compared with usual care. Qualitative and fidelity sub-studies will be analysed through framework analysis and fidelity assessment tools respectively.

Ethics and dissemination

REC and HRA approval were obtained. A Data Monitoring and Ethics Committee has been constituted. Dissemination will be via publications, conferences and social media. Intervention materials will be made open access.

Trial registration number

ISRCTN17993825

For peer review only

STRENGTHS AND LIMITATIONS OF THE STUDY

- People living with dementia were involved in developing the content of the Journeying through Dementia (JtD) intervention and are involved in advising the study.
- The JtD intervention includes sessions without supporters present, to help develop independence and confidence, and is one of the only interventions that people with dementia can participate in without supporters.
- The JtD study will recruit up to 500 participants and is therefore one of the largest trials of a psychosocial intervention for people with dementia in the UK.
- The potential for unblinding of researchers when arranging or attending follow-up visits is a limitation but this will be monitored and minimized by not sending unblind researchers to visits.
- Recruitment is known to be challenging in this population but we plan to try multiple pathways for recruitment including through services, the Join Dementia Research database, promotion and the third sector.

For peer review only

INTRODUCTION

The impact on the economy, for services and for individuals living with dementia and their family carers is larger for dementia than for all other long-term illnesses in people aged 60 and over.[1] Two thirds of people with dementia live in the community, and half of these require some form of support.[2] As a result, dementia research (both for cure and for care) in the NHS and social care is important, and this is reflected in UK health policy.[3] In 2009, the UK Government announced a National Dementia Strategy, which mandated the establishment of memory services and aimed to increase the rates of early diagnosis and improve support for people in the early stages of dementia.[4]

The National Audit of Memory Services (2013) found there had been a fourfold increase in numbers presenting since 2010/11, and in 2013 49.3% were in the early stages of the condition.[5] Earlier diagnosis allows individuals to receive treatment earlier and enables the individual and memory services to plan more effectively for the future.[6] Memory services have been strongly encouraged to provide post diagnostic treatment and support,[7,8] but the availability of appropriate evidence-based interventions has not kept pace with the increase in demand, in particular for those with early stages of dementia. The lack of appropriate interventions has led to inconsistency between Trusts regarding what is being offered to people post diagnosis.

The potential value of psychosocial interventions for people in the early stages of dementia is recognised[9–12] and is also driven by the knowledge that a cure for dementia is unlikely in the near future. Psychosocial interventions are diverse but a common theme is that they do not involve the use of medication and instead focus on supporting people to overcome challenges and maintain independence and well-being. However, whilst there has been some shift, the use of psychosocial interventions within dementia care has been a neglected area for both research and practice.[11]

There is a growing body of evidence to demonstrate how individuals with dementia can be supported to use self-management based techniques (sometimes in combination with other interventions such as cognitive rehabilitation and occupational therapy).[13–18] A qualitative study

of people with dementia who attended a self-management programme reported that participants identified the opportunity for peer support as being beneficial and considered that the programme could be improved by greater emphasis being placed upon maintaining activities and relationships and improving positive wellbeing.[19] The Healthbridge evaluation[20] and the Mental Health Foundation evaluation[21] found evidence that people with dementia and their carers can benefit from receiving group based peer support.

The Journeying through Dementia (JtD) intervention was developed from the Lifestyle Matters programme and a pilot study was conducted to examine the feasibility of a future population-based larger trial of this intervention.[22] The intervention was found to be acceptable to both people with dementia and their carers. Reported benefits included increased confidence and self-efficacy, engagement in activities and re-engagement with fun and friendships.[22] The intervention is manualised, based on occupational therapy principles, and is designed to support independence and well-being. The intervention incorporates elements of self-management and group-based peer support and has been designed to improve the quality of life for people in the early stages of dementia.

The JtD randomised controlled trial (RCT) will test the clinical and cost effectiveness of the JtD intervention. Funding was obtained through the National Institute of Health Research (NIHR) Health Technology Assessment (HTA) theme to conduct the RCT. This paper describes the research protocol for undertaking the RCT which started recruitment in November 2016.

AIMS AND OBJECTIVES

The primary aim of the JtD trial is to determine the clinical and cost-effectiveness of the JtD intervention for people in the early stages of dementia. To meet this aim, the objectives are to:

1. Conduct an internal pilot RCT of the intervention to check the feasibility of rates of recruitment at scale.
2. Proceed to a full a pragmatic RCT evaluating the clinical and cost-effectiveness of the JtD intervention.
3. Conduct fidelity checks regarding the delivery of the JtD intervention.
4. Undertake an embedded qualitative sub-study to explore issues concerned with intervention delivery.
5. Identify how the intervention might be realistically delivered through services.

METHODS

Trial design

The JtD trial is a pragmatic, two-arm, parallel group, individually randomised RCT. It uses a superiority framework to deliver an intention-to-treat comparison of the JtD intervention with usual care.

The trial includes three sub-studies. The first is a health economics evaluation using a cost-effectiveness analysis of the incremental cost per Quality Adjusted Life Year (QALY) of the JtD intervention compared with usual care.

For peer review only - http://bmjopen.bmj.com/site/about/guidelines.xhtml

The second sub-study is a fidelity assessment as part of the process evaluation, using an intervention fidelity framework based on that identified by the Behaviour Change Consortium and National Institute for Health and Care Excellence (NICE).[23,24]

The third sub-study is a qualitative study, part of the process evaluation, in line with Medical Research Council (MRC) guidance on developing and evaluating complex interventions.[25] The qualitative sub-study will involve semi-structured interviews with participants, supporters, facilitators and supervisors.

Randomisation, blinding and bias

To minimise bias, allocation will be concealed through the use of a centralised web-based randomisation service. The randomisation sequence will be stratified by delivery site and constrained by a fixed block size to ensure participants are allocated evenly to each arm of the trial at each delivery site. Participants will be randomised in equal numbers to intervention and control arms. An unblind member of the research team who will not be conducting outcome assessments will enter the participants' details into the randomisation system and inform the relevant parties of the outcome.

Members of the Trial Steering Committee (TSC), study statisticians, health economists and outcome assessors will be blinded to treatment allocation whilst the trial is ongoing. For practical reasons linked to the provision of a centralised web-based randomisation service and the set-up and delivery of the intervention groups, some members of the research team will not be blinded, including the Trial Manager and Chief Investigator. Due to the nature of the intervention, participants will not be blinded. If the outcome assessors know (or suspect) they have been unblinded this will be recorded on an unblinding form.

We protect against facilitator bias where the same facilitators also provide usual care in three ways: (a) usual care is limited and often restricted to cognitive stimulation therapy which would not be readily influenced by training in JtD principles, as it follows a manual with a session-by-session plan already laid out; (b) where usual care was also delivered by intervention facilitators, they were instructed not to deliver JtD wholly or partly as part of usual care; (c) many of the facilitators recruited are not those who would deliver usual care, for example approximately 25% of facilitators are trained research staff. A further form of bias, that caused by cross-contamination of participants between the two study arms, is considered as unlikely as post diagnostic services for people living with dementia are limited and often only involves cognitive stimulation therapy which is unlikely to be concurrent. Extended post diagnostic follow-up is not common so it is unlikely participants would meet at routine appointments.

Participants

Persons with early-stage dementia will be approached to take part in the trial. A family member, friend or neighbour that provides support to the study participant (referred to as the 'supporter') can be approached to take part in the trial, but only if invited to do so by the person with dementia. The study participant can also choose not to invite a supporter to take part in the trial and still take

part. Participating supporters will automatically be randomised to the same arm as the participant. See Figure 1 for participant flow through the study.

(Insert Figure 1 here)

Eligibility criteria

Participants will be eligible for the study if they:

1. have a diagnosis of any form of dementia,
2. have a Mini Mental State Examination (MMSE) score of 18 or more, measured less than 2 months' pre-consent,
3. have capacity to make informed decisions,
4. are living in the community in their own or sheltered accommodation (those living in residential or nursing care are not eligible),
5. are willing to attend the JtD intervention,
6. are able to converse and communicate in English, and
7. are not taking part in any other pharmacological or psychosocial intervention studies at the time of enrolment.

Supporters will be eligible to participate if they are aged 18 years or over, are named by the person with dementia as their supporter, the person with dementia wishes them to take part, they can converse and communicate in English, and have the ability to give informed consent.

Site selection and participant recruitment

The JtD trial will operate in England within 13 NHS trusts with specialist dementia services. The sites will be selected based on a convenience sample of locations clustered geographically around the north and midlands (for a list of sites, see study ISRCTN webpage[26]).

Previous studies involving persons with dementia have shown that recruiting participants to such studies can be challenging.[27] To ensure sufficient numbers are recruited to the study a number of recruitment pathways will be used: referral from clinical teams; mail-outs to eligible patients via primary or secondary care clinical teams; study promotion via posters etc.; third sector organisations; and the Join Dementia Research database. The research team will contact interested participants and send further information about the study. Clinical teams will check with the potential participants that they are willing to be approached by the research team before an approach is made.

To enable potential participants to make a direct approach to the research team, a reply card will be designed which can be completed, sealed and returned to the central research team who will then pass the information on to relevant local site researchers. These reply cards will be distributed with information sheets at events including dementia cafes or posted as part of mail-outs to GPs. Local promotions, for example via posters in clinics or GPs, will include the telephone and email details of local researchers for direct contact to take place.

A consent and eligibility visit will be arranged if an individual would like to take part where trained researchers will assess eligibility and request written informed consent.

For peer review only - http://bmjopen.bmj.com/site/about/guidelines.xhtml

Intervention

JtD is a manualised intervention consisting of twelve weekly facilitated groups with 8-12 participants with dementia, which take place over successive weeks. Each participant also receives four one-to-one sessions with one of the intervention facilitators, to pursue individual goals. The first one-to-one session takes place before the start of the group intervention, with the remaining three being scheduled during, the 12 weeks and at locations and times agreed with the participant. The group aspect of intervention should be delivered in a community venue.

The content of the JtD intervention involves:

a. Ways of thinking about dementia (what is dementia, effects on everyday life, challenging stereotypes, sharing coping strategies).
b. Keeping physically well (relationship between physical and mental wellbeing, embedding health activity in everyday life, diet).
c. Memory (strategies to aid memory, impact on everyday life and learning and practicing new techniques).
d. Keeping mentally well (relationships between anxiety and memory, and dementia and stress).
e. Endings (celebration of achievements and how to move forward).

Participants are encouraged to select different topics from the manualised intervention and explore them with guidance and suggestions from facilitators. Participants are also able to suggest topics not within the manual. One essential component of the intervention is the enactment of activities, particularly in the community; three of the twelve group meetings should be 'out of venue' activities. Participants are able to invite a supporter to participate in the group aspect of the intervention during sessions 1, 6 and 12, and in the individual sessions if the participant finds this helpful in achieving their goals.

The intervention should be facilitated by a minimum of two NHS staff who are experienced with working with people with dementia. Facilitators will normally be someone employed on Agenda for Change Bands 3-5. Facilitators must receive a two-day training course prior to delivering the intervention. In some cases, for example if they are a reserve facilitator, they may receive a shortened course supported by online resources created for this purpose. Facilitators will be supervised by a colleague experienced in supervision who has also attended the facilitator training and is of suitable seniority and experience. The supervisors themselves will be supervised by a member of the central study team who is a Clinical Psychologist and is experienced in the 'train the trainer' method[28].

Patient and public involvement (PPI)

People living with dementia were involved in developing the content of JtD[29] and in the feasibility study.[22] The JtD Trial Steering Committee will include a member who is living with dementia. Additionally, an advisory group of people living with dementia (experts by experience) will meet at intervals throughout to provide input into study materials. We also plan to involve people living with dementia in some aspects of the qualitative data analysis and in creating and delivering the study dissemination plans.

For peer review only - http://bmjopen.bmj.com/site/about/guidelines.xhtml

OUTCOMES

Outcomes were identified through a feasibility study which identified appropriate measures and tested their application.[22] The burden of questionnaires was found to be acceptable.

The primary outcome is the Dementia Related Quality of Life (DEMQOL)[30,31] measure at 8 months post randomisation.

The secondary outcomes are:

- European Quality of Life - 5 Dimensions (EQ-5D-5L)[32,33]
- Patient Health Questionnaire-9 (PHQ-9)[34,35]
- Generalised Anxiety Disorder-7 (GAD-7)[36]
- General Self-Efficacy Scale (GSE)[37]
- Diener's Flourishing Scale (DFS)[38]
- Self-Management Ability Scale (SMAS)[39]
- Instrumental Activities of Daily Living (IADL)[40]
- Health and Social Care Resource Use Questionnaire (HSCRU)[22]
- Sense of Competency Questionnaire (SCQ)[41],[42]

See Table 1 (below) for further information on who completes which measures in the study. The outcome measures used were selected to measure the following key components of the intervention: mental wellbeing or mood (DEMQOL, PHQ-9, GAD-7); building relationships and a sense of connectedness (DFS, SMAS); self-management (SMAS); belief that life is meaningful despite dementia (DEMQOL, GSE, DFS, SMAS); and Instrumental Activities of Daily Living (IADL) and strategies to maintain cognitive functioning (IADL). Additionally, they also support analysis of the cost-effectiveness of the intervention (DEMQOL, EQ-5D-5L, HSCRU) and the participating supporters' perceptions of competence (SCQ). Dementia specific outcome measures are those recommended for use across Europe and are selected for self rather than proxy completion.[43] Non dementia specific outcome measures were selected if there was no appropriate dementia specific measure available.

DATA COLLECTION

Quantitative

Data will be collected from all participants living with dementia at eligibility/consent, baseline, 8 and 12 months post randomisation and from consented participating supporters at baseline and 8 months' post randomisation (see Table 1). There is a reduced set of measures linked to the 12 month visit as we require limited further information on quality of life and health and social care resource use at that time-point; the key outcome point is at 8 months. The outcome measures will be interviewer-administered at face-to-face visits by blinded outcome assessors who have received training to deliver the measures to people living with dementia and their supporters. The follow-up outcome measures will be collected 2 weeks pre and 8 weeks post the date they are due. Participant retention will be promoted by regular communication with the participants and supporters through communication including newsletters and Christmas cards.

Table 1 Outcome measures and time-points for collection. * Denotes the primary outcome measure.

For peer review only - http://bmjopen.bmj.com/site/about/guidelines.xhtml

Measure	Participant				Participating Supporter	
	**Eligibility and Consent visit**	**Baseline** <2 months prior to the intervention start date	**8 Months** Due < 2 weeks pre and < 8 weeks post randomisation	**12 Months** Due < 2 weeks pre and < 8 weeks post randomisation if within study timelines	**Baseline** Due <2 months prior to the intervention	**8 Months** Due < 2 weeks pre and < 8 weeks post randomisation
Capacity assessment	✓					
Mini Mental State Examination	✓					
Eligibility checklist	✓				✓	
Baseline demographics	✓				✓	
DEMQOL		✓	✓*	✓		
EQ-5D-5L		✓	✓	✓	✓	✓
PHQ-9		✓	✓		✓	✓
GAD-7		✓	✓			
GSE		✓	✓			
DFS		✓	✓			
SMAS		✓	✓			
IADL		✓	✓	✓		
HSCRU			✓	✓		
SCQ					✓	✓

Visits to collect outcome data will be arranged with the participant by a researcher, in some cases a participating supporter will assist with these arrangements. All visits will be conducted at a time and location most suitable for the participant. When we conduct the follow-ups we will prioritise the importance of the measures in case the participant tires. We will offer a second visit if the participant is tired or otherwise unable to complete the assessments, which will be organised as soon as possible after the first. Participating supporters may not have the time or capacity to receive a face-to-face visit and follow-up outcome measures may therefore be collected from them over the telephone. Similarly, if a participant does not want a visit, a reduced set of outcome measures may be taken by telephone (prioritising collection of DEMQOL and telephone versions of HSCRU and EQ-5D-5L).

Intervention attendance

Records will be kept of all attendances for each participant randomised to the intervention.

Intervention dropout and study withdrawal

If a participant decides to withdraw either from the intervention or the study, this will be recorded. If the participant just withdraws from the intervention, they will be followed-up unless they explicitly also withdraw consent for follow-up meetings for collection of outcomes (data up to this time will be included in the trial). If the participant fully withdraws from the study no further data will be collected.

For peer review only

For peer review only - http://bmjopen.bmj.com/site/about/guidelines.xhtml

Intervention costings

Information on the cost of facilitated group and individual sessions will be collected including hire of local community venues, facilitator salaries and travel, refreshments, and other costs such as administration and materials used.

SAMPLE SIZE

The primary outcome for the study is the mean DEMQOL score 8 months post randomisation. Assuming a standard deviation of 11 points for the DEMQOL, a mean difference of 4 or more points is clinically and practically important.[30] The sample size has been calculated to have a 90% power of detecting this 4 point difference (equivalent to a standardised effect size of 0.36) in group mean scores at eight months as being statistically significant at 5% (two sided) level. As the JtD intervention is a facilitator led intervention with a group component, the outcomes of the participants in the same group with the same facilitators may be clustered. With no adjustment for clustering by facilitator the target sample size would be 160 per arm with a total sample size of 320. We have assumed an average cluster size of 8 people with dementia per facilitated group and an intra-cluster correlation of 0.03; this will inflate the sample size by a design effect of 1.21; to 194 per group (388 total sample size) with valid primary outcome data. Assuming at least a 20% loss to follow-up the target sample size for the trial is to randomise to 243 participants in each arm (n=486).

DATA ANALYSIS

Statistical data analysis

As JtD is a pragmatic parallel group randomised trial, with a usual care (control) arm, data will be reported and presented according to a revised CONSORT statement.[44] Statistical analysis will be performed on an intention-to-treat-basis. All exploratory tests will be two-tailed with alpha = 0.05. Baseline demographics and quality of life data will be described and summarised overall and by treatment group.

The primary analysis will compare mean patient reported DEMQOL scores at 8 months post randomisation between the intervention (JtD) arm and control arms using a mixed effects linear regression model adjusted for DEMQOL baseline score and site and allowing for the clustering of the outcome by the JtD intervention.[45–47] The trial is a partially nested design with comparison of a group therapy (JtD) with individual therapy with clustering in one (intervention) arm. Each person with dementia in the control group (unclustered arm) will be treated as a cluster (singleton) of size one. The cluster indicator will be treated as a random effect. A stratification variable used for randomisation (site) will be included as a fixed factor.[48] A partially clustered mixed effects linear regression model with homoscedastic errors as well as a heteroscedasticity mixed effects linear regression model will also be considered to account for potential differential variability of outcomes between the two treatment groups. A 95% confidence interval (CI) for the mean difference in DEMQOL scores between the intervention and control groups will be calculated together with the associated P-value. A further adjusted analysis may also be performed depending on the observed degree of imbalance in baseline covariates (which are of potential prognostic importance) again using a mixed effects linear regression model. Additional covariates (of potential prognostic importance) include other baseline variables, such as age, gender, PHQ-9, and GAD-7. In the event

For peer review only - http://bmjopen.bmj.com/site/about/guidelines.xhtml

that there are more than 10 couples (20 participants) living under the same roof from different households in the study, then the primary and secondary analyses will be changed to take into account the hierarchical or clustered nature of the data. A multi level mixed effects model will be used; the random effects will be JtD intervention groups (top level) and couple/singles (lower level). Individual participants who are not part of a couple will be treated as clusters of size one.

Participants will be followed up for up to 12 months post randomisation. Mean DEMQOL scores at 12 months follow-up will be compared as described for the primary outcome above.

For the primary outcome, the DEMQOL score at 8 months follow-up, missing data will be imputed through a variety of methods including: regression and multiple imputation as part of a sensitivity analysis.

We will complement the intention to treat (ITT) analysis of the primary outcome with a complier average causal effects analysis (CACE) as a secondary analysis alongside the primary ITT analysis. Compliance will be defined as a binary variable with participants who attend at least 10 of the 16 JtD sessions (both individual and group sessions combined) regarded as being compliant.

There are no planned interim statistical analyses or formal stopping rules in relation to efficacy.

In terms of missing data, the primary analysis will be performed based on participants with available 8 month primary outcome data. Sensitivity analysis on the primary outcome will include multiple imputation using chained equations and regression imputation.

Secondary outcome measures

Secondary outcomes at 8 and 12 months post-randomisation will be compared between the intervention and control groups using a mixed effects linear regression model as for the primary outcome. A 95% CI for the mean difference in this parameter between the treatment groups will also be calculated together with the associated P-value.

Outcome measures for the participating supporters at 8 months will be compared between the intervention and control groups using a mixed effects linear regression model. The mean difference in outcome with associated 95% CI and P-value will be presented for: a) the baseline (specific to the secondary outcome) and site adjusted analysis and b) adjusted analysis with additional covariates in addition to a).

Subgroup analysis

A subgroup analysis using a mixed effect linear regression model, with the primary outcome (DEMQOL) at 8 months post randomisation as the response will be carried out. We will use an interaction statistical test between the randomised intervention group and subgroup to directly examine the strength of evidence for the treatment difference between the treatment groups varying between subgroups. Supporter involvement (yes or no) will be the only a priori defined sub groups to be considered for interaction test.

HEALTH ECONOMICS EVALUATION

For peer review only - http://bmjopen.bmj.com/site/about/guidelines.xhtml

A trial based economic evaluation will be undertaken of an intention-to-treat comparison of the costs and outcomes of the two trial arms. A cost-effectiveness analysis will be undertaken of the incremental cost per Quality Adjusted Life Year (QALYs) of the JtD intervention compared with usual care provided through NHS memory services. QALYs will be calculated using the EQ-5D-5L preference-based index administered at baseline, 8 and 12 months. A sensitivity analysis will be undertaken using utility values from the DEMQOL-U, which can be derived from responses to the DEMQOL questionnaire.[30] The total cost of the intervention will be estimated at the individual participant level and will include the costs of providing the intervention and the subsequent consequences for the use of routine health and social care services. The average cost per attendance will be calculated and this estimate will be applied to the actual number of group and individual sessions that each participant attended.

The use of services by trial participants will be collected in detail using a Health and Social Care Resource Use (HSCRU) questionnaire administered at 8 and 12 months post randomisation. Service use will be costed using the most recent National Reference Cost Data and Unit Costs of Health and Social Care.[49,50] Missing data will be dealt with using multiple imputation for EQ-5D-5L, DEMQOL-U and resource use data.[51] A random effects linear regression model, accounting for clustering, will be fitted, the model will include baseline scores for EQ-5D-5L and baseline costs. The central analysis of mean incremental costs per QALY will be subjected to a full sensitivity analysis of key parameters including the measure used to estimate QALYs and number of participants at the weekly sessions. A full probabilistic sensitivity analysis will be performed to examine the probability of cost-effectiveness of the intervention for the NHS for different levels of costs and QALY gains.[52]

PROCESS EVALUATION

The process evaluation has been designed to: 1) examine the factors that may influence the fidelity of intervention delivery and 2) explore its perceived impact and acceptability from user and provider perspectives. There are two strands to this, the fidelity assessment and qualitative interviews, explained below.

Fidelity assessment

The fidelity of intervention delivery, as well as of the training and supervision received by facilitators will be assessed on the basis of criteria derived from the intervention protocol and manual. Fidelity checks will adhere to a framework based on that identified by the Behaviour Change Consortium[23] and NICE guidance on behaviour change.[24] The fidelity assessment framework (adapted from Bellg et al, 2004[23]) will assess and monitor for the consistency, facilitator training (in terms of standardised delivery of training and skills acquisition for facilitators), intervention delivery (in terms of standardised delivery between intervention groups) and the receipt of the intervention by intervention group participants.

Training delivery and receipt of the facilitator training will be observed and rated by the same two researchers (the lead for fidelity and one other member of the research team) for inter-rater reliability using a bespoke *Training observation checklist*. To assess facilitator adherence to the manualised intervention and participant receipt of the intervention, a purposive selection of group meetings across sites will also be observed using a *Group observation checklist* which is based on the contents of the intervention and the training. Each selected groupwill be observed on two occasions

For peer review only - http://bmjopen.bmj.com/site/about/guidelines.xhtml

to identify facilitator drift or changes in participant behavior.

Frequencies will be used to determine the extent to which the training programme received by facilitators maintained fidelity to what was intended. Data will also be analysed to compare intervention delivery between and across sites to check for consistency. Inter-rater reliability between coders will be determined using the Kappa statistic.[53] Similar methods have been used in previous studies.[54]

Qualitative interviews

An embedded qualitative sub-study will explore the mechanisms of the intervention, for example what elements of the intervention appear to support people to improve their self-management and well-being and what promotes good facilitation of the intervention.

Individual qualitative semi-structured interviews will be conducted with a purposive sample of 20 participants (approximately 10%) from the intervention groups observed in the fidelity sub-study and with approximately 12 participating supporters, preferably supporters of participants who are also being interviewed. A participant interview schedule and supporter interview schedule will be developed to cover the following themes:

- Range and nature of issues that influence experiences of taking part in the intervention
- Factors that may influence the effectiveness, adoption and diffusion of this innovation in the future
- Perceived skills and competencies required to facilitate the intervention
- The barriers and facilitators to uptake and continued use
- The effect of the intervention upon living with dementia
- The effect of the intervention upon the experiences of supporting someone with dementia

Semi-structured interviews will also be conducted with approximately 20% of all facilitators and supervisors across the sites upon completion of their delivery of the intervention. The sample will include a range of sites and facilitators with different levels of experience of delivering the intervention. A facilitator interview schedule and supervisor interview schedule will be created to cover the following themes:

- What issues promote the effectiveness of intervention facilitation
- The skills and competencies required to facilitate the programme
- The barriers and facilitators to its uptake and continued use
- Factors that may mediate or moderate the effectiveness of the intervention

Researchers undertaking participant interviews will be trained to use enhanced methods of communication with people with dementia to try to ensure that meaningful discussion takes place. Transcripts of interviews will undergo respondent validation.

Qualitative analysis

Framework analysis[55] will be applied to all interview data.[56] For the purposes of reporting, confidentiality will be maintained by using unique participant identifiers and removing identifiable

For peer review only - http://bmjopen.bmj.com/site/about/guidelines.xhtml

information. A thematic framework will be agreed by two researchers and an index developed for transcript coding. This will follow the five stages of framework analysis.[55] Findings will be used to identify emergent factors that influence the uptake and impact of the intervention as well as explore potential explanations for the quantitative findings.[56] Further analysis will also be undertaken to triangulate the qualitative data (between facilitator/supervisor and participant/supporter interviews) as well as the fidelity and qualitative data in order to look for between source similarities and divergences. Analysis workshops will be held with people living with dementia and their supporters to respond to and help validate the initial qualitative analysis. The workshop outcomes will be used to refine the qualitative analysis.

DATA MANAGEMENT, CONFIDENTIALITY AND SHARING

Sheffield Clinical Trials Research Unit (CTRU) will provide data management services. Data will be entered remotely on to a centralised web-based data capture system (Prospect) by university researchers and authorised staff at participating NHS sites. The Case Report Form captures trial data and has been specifically designed for this trial. Access to Prospect is controlled by usernames and encrypted passwords. Prospect provides a full electronic audit trail, as well as validation features which will be used to monitor study data quality. The identity of participants will be protected by the removal of any identifiable data prior to dissemination of information, and no identifiable data will be transferred to the statistician or health economist. All participating NHS Sites will be subject to data monitoring reviews to check data entry, consent and eligibility, amongst other items. The trial follows the UK Health Research Authority guidance on the General Data Protection Regulation[57] and has implemented a privacy policy and transparency information appropriate to people living with dementia.

Data sharing

JtD trial data will be held and available for five years after the end of the trial (November 2019). JtD Trial Data will not be archived in a repository, instead data will be released on a case-by-case basis. We shall make data available to the scientific community with as few restrictions as feasible. Data access requests will be reviewed and authorised by a sub-committee of the Trial Management Group (TMG) during the trial and by the Sheffield CTRU after the trial has ended. Access requests will be considered against pre-determined criteria and data sharing will only take place if this aligns with the consent provided by JtD participants. Data will be anonymised prior to being shared.

ETHICS, GOVERNANCE AND SAFETY

Ethical issues

There are two key ethical issues to take into account. The first is the potential need to break confidentiality where there is a risk of harm. We will request consent to contact the participants or supporter's GP or other health professional in situations where researchers are concerned that there might be risk of harm. For example, two outcome measures used on the study may indicate a need to clinically treat anxiety (GAD-7) or depression (PHQ-9). Additionally, concerns regarding participant safety may also be raised at any stage of the study; e.g. observed deterioration in mental or physical state of participants, safeguarding issues, or of a risk to self or others. We will work alongside local

For peer review only - http://bmjopen.bmj.com/site/about/guidelines.xhtml

Trust procedures to report any risks appropriately. Local site investigators with clinical backgrounds will be asked to provide advice when appropriate. The responsible healthcare professional or relevant PI will be able to recommend the withdrawal of the participant if they feel it is appropriate. We will record all actions taken.

The second is the risk that during the trial, people with dementia may lose the capacity to consent to continuing participation. As part of the consent process, people with dementia will be asked to nominate a person to act as a consultee we may contact in the event that they lose capacity during the trial. The consultee will be independent from the study and can be a relative, friend or medical professional. All researchers will be provided with guidance and local training on identifying and dealing with capacity issues identified before or during 8 and 12 month follow-up visits. If at any point the participant indicates they do not wish to continue to take part in the trial, they will be withdrawn. If the person with dementia loses capacity during the trial, but indicates they wish to remain in the study, the consultee will be asked to make a judgement, based on their existing and pre-existing knowledge of the person with dementia, about whether they would want to continue participation or not. If the consultee advises that the participant would wish to be withdrawn, the researchers will withdraw them from the study. If the consultee advises that the participant would wish to stay taking part, even though they lack capacity, every effort will be made to accommodate this, for example by reducing the number of outcome measures, using prompts or other means to help the participant complete the measures. We will record information about the activation of the consultee pathway on the trial.

Governance

The trial is coordinated by the Sheffield CTRU on behalf of the Sponsor. The sponsor of the trial is Sheffield Health and Social Care Foundation Trust, Fulwood House, Old Fulwood Road, Sheffield, S10 3TH. The JtD TMG contains project co-applicants, members of the data management team, the Sponsor, Trial Manager and other representatives, and oversees the operation of the trial and enables communication throughout the Trial, for example to disseminate protocol amendments. An independent Trial Steering Committee (TSC), comprised of an independent statistician, PPI representative and a Senior Clinical Research Associate, provides overall supervision of the trial, advises the CI, oversees protocol modifications, monitors the trial's progress and if necessary closes the trial. An independent Data Monitoring and Ethics Committee (DMEC) comprised of two independent statisticians and an Occupational Therapist Clinical Researcher, reviews the trial protocol, monitors patient safety and advises the TSC if they feel the trial should be prematurely closed.

Safety

A Serious Adverse Event (SAE) reporting system will be used on the Trial. Adverse Events are not anticipated as a consequence of the intervention and will not be monitored. An SAE either:

a) results in death,
b) is life-threatening (subject at immediate risk of death),
c) requires hospitalisation or prolongation of existing hospitalisation,
d) results in persistent or significant disability or incapacity, or

For peer review only - http://bmjopen.bmj.com/site/about/guidelines.xhtml

e) is otherwise considered medically significant by the investigator.

All SAEs will be assessed to see if they are related to the intervention or other trial procedures, and if they are the Sponsor and Research Ethics Committee will be immediately informed. SAEs are periodically reported to the trial's DMEC.

Additionally, we consider safety of the researchers to be extremely important and have developed a lone worker policy. The researcher must complete a form detailing information about any participant visits and their contact information and provide this to a 'buddy' who will ensure the safety of the researcher. The researcher must check in with the buddy before a visit and after a visit finishes or the buddy will follow escalation procedures. Check-lists provide guidance on what to do before and during the visits, for example ensuring phones are fully charged and being prepared to leave in an emergency if there are concerns about safety. A phrase is provided to enable the researcher to report an emergency during the visit. Guidance is provided for general safe travelling, for example to keep to well-lit paths and driveways.

DISSEMINATION

The results of this study will be communicated in relevant academic and professional journals, conferences and workshops, and via websites and social media, ensuring reach to all stakeholders (people living with the condition, professionals, commissioners and academics). A short film will be created to illustrate experiences of participation from the perspectives of people living with the condition, and those involved in delivery of the intervention. The manualised intervention will be refined and made available on the website in an open access format and it is anticipated that this may be of interest to both primary care and memory services.

AUTHOR CONTRIBUTIONS

GM, AF, CCo, KS, EL, SW, TY, CCr, EMC, AL, TD and KB co-wrote the original Trial Protocol. JW led the development of the Protocol for publication. JBD and KS developed the sections on fidelity and qualitative analysis. JBD developed the section on PPI. BDT adapted the introductory section, BJT the methods section, EY the outcomes section and EL the statistical analysis section. All authors contributed to reviewing and revising the draft versions prior to submission.

ACKNOWLEDGEMENTS

We would like to thank the following people for assisting with the original project application: Martin Orrell, Institute of Mental Health, The University of Nottingham; Daniel Blackburn, Sheffield Institute for Translational Neuroscience, The University of Sheffield; and Diana Papaioannou, Sheffield Clinical Trials Research Unit, School of Health and Related Research, The University of Sheffield.

Additionally, we would like to thank Peter Bowie, Old Age Psychiatry, Sheffield Health and Social Care NHS Foundation Trust, Sheffield, for advising on clinical aspects; and Nicholas Bell, Sheffield Health and Social Care NHS Foundation Trust, for acting on behalf of the sponsor and advising on trial procedures.

FUNDING STATEMENT

For peer review only - http://bmjopen.bmj.com/site/about/guidelines.xhtml

This study was funded by the NIHR Health Technology Assessment Programme (project number 14/140/80). The views expressed are those of the author(s) and not necessarily those of the NHS, the NIHR or the Department of Health.

COMPETING INTERESTS

All authors, with the exception of those below, declare no competing interests.

- SW, TY, CCo, JW, AF, AL, EL, BJT, BDT, EY as ScHARR has contracts and/or research grants with the Department of Health, NIHR, MRC and NICE.
- SW, CCo and TY are co-applicants or co-investigators on NIHR portfolio grants (NIHR Research Design Service for Yorkshire and Humber; HTA, RfPB, PHR; SDO) and grants from the MRC.
- SW and TY also receive external examining fees from various UK higher education institutes.
- SW receives book royalties from publishers including John Wiley and Sons Ltd and Blackwell Publishing.
- BDT is a Consultant for Arch research in the area of dementia.

ETHICS APPROVAL

This study was approved by Leeds East Research Ethics Committee, on 01/07/16, reference number 16/YH/0238. Health Research Authority approval was provided for the study to commence on 25/08/16. The current Protocol version is v7, 5th December 2018.

REFERENCES

1 World Health Organisation. The global burden of disease: 2004 update. Geneva: World Health Organisation 2008.

2 Knapp M, Comas-Herrera A, Somani A, *et al.* Dementia: international comparisons – summary report to the National Audit Office. London: London School of Economics 2007.

3 Department of Health. Prime Minister's challenge on dementia 2020. London: Department of Health 2015. https://www.gov.uk/government/uploads/system/uploads/attachment_data/file/406076/Dementia_vision.pdf (accessed 26 Sep 2018)

4 Department of Health. Living well with dementia : A National Dementia Strategy. London: Department of Health 2009. https://www.gov.uk/government/publications/living-well-with-dementia-a-national-dementia-strategy (accessed 26 Sep 2018)

5 Hodge AS, Hailey E. English National Memory Clinics Audit Report. London: Royal College of Psychiatrists 2013. http://www.rcpsych.ac.uk/pdf/English National Memory Clinics Audit Report 2013.pdf (accessed 30 Oct 2018)

6 Banerjee S, Wittenberg R. Clinical and cost effectiveness of services for early diagnosis and intervention in dementia. *Int J Geriatr Psychiatry* 2009;24:748–54. doi:10.1002/gps.2191

7 Copland E, Hodge S, Clary L, *et al.* Memory Services National Accreditation Programme (MSNAP): Standards for memory services. London: Royal College of Psychiatrists 2018. https://www.rcpsych.ac.uk/pdf/MSNAP_Standards_6_Edition_2018.pdf (accessed 16 Oct 2018)

For peer review only - http://bmjopen.bmj.com/site/about/guidelines.xhtml

8 NHS England. Well Pathway for Dementia. 2017. https://www.england.nhs.uk/mentalhealth/wp-content/uploads/sites/29/2016/03/dementia-well-pathway.pdf (accessed 30 Oct 2018)

9 National Institute for Health and Clinical Excellence. Donepezil, galantamine, rivastigmine, and memantine for the treatment of Alzheimer's Disease. London: NIHCE 2011. http://www.nice.org.uk/guidance/ta217/resources/guidance-donepezilgalantaminerivastigmineandmemantine-for-the-treatment-of-alzheimers-disease-pdf (accessed 26 Sep 2018)

10 Woods B, Aguirre E, Spector AE, *et al.* Cognitive stimulation to improve cognitive functioning in people with dementia. *Cochrane Database Syst Rev* 2012;15. doi:10.1002/14651858.CD005562.pub2

11 Moniz-Cook E, Vernooij-Dassen M, Woods B, *et al.* Psychosocial interventions in dementia care research: The INTERDEM manifesto. *Aging Ment Health* 2011;15:283–90. doi:10.1080/13607863.2010.543665

12 McDermott O, Charlesworth G, Hogervorst E, *et al.* Psychosocial interventions for people with dementia: a synthesis of systematic reviews. *Aging Ment Health* Published Online First: 2018. doi:10.1080/13607863.2017.1423031

13 Clare L, Woods RT. Cognitive training and cognitive rehabilitation for people with early-stage Alzheimer's disease: A review. *Neuropsychol Rehabil* 2004;14:385–401. doi:10.1080/09602010443000074

14 Graff MJL, Vernooij-Dassen MJM, Thijssen M, *et al.* Community based occupational therapy for patients with dementia and their care givers: randomised controlled trial. *BMJ* 2006;333:1196. doi:10.1136/bmj.39001.688843.BE

15 Vernooij-Dassen M, Olde Rikkert MG. Personal disease management in dementia care. *Int J Geriatr Psychiatry* 2004;19:715–7. doi:10.1002/gps.1149

16 Moniz-Cook E, Manthorpe J, eds. Early Psychosocial Interventions in Dementia. London: Jessica Kingsley Publishers 2008.

17 Quinn C, Toms G, Anderson D, *et al.* A review of self-management interventions for people with dementia and mild cognitive impairment. *J. Appl. Gerontol.* 2016;35:1154–88. doi:10.1177/0733464814566852

18 Quinn C, Toms G, Jones C, *et al.* A pilot randomized controlled trial of a self-management group intervention for people with early-stage dementia (The SMART study). *Int Psychogeriatr* 2016;28:787–800. doi:10.1017/S1041610215002094

19 Martin F, Turner A, Wallace LM, *et al.* Qualitative evaluation of a self-management intervention for people in the early stage of dementia. *Dementia* 2015;14:418–35. doi:10.1177/1471301213498387

20 Clarke CL, Keyes SE, Wilkinson H, *et al.* Healthbridge: The National Evaluation of Peer Support Networks and Dementia Advisers in Implementation of the National Dementia Strategy for England. London: Department of Health 2013.

21 Chakkalackal L, Kalathil J. Evaluation report: Peer support groups to facilitate self-help coping strategies for people with dementia in extra care housing. London: Mental Health Foundation 2014.

For peer review only

22 Sprange K, Mountain GA, Shortland K, *et al.* Journeying through Dementia, a community-based self-management intervention for people aged 65 years and over: a feasibility study to inform a future trial. *Pilot Feasibility Stud* 2015;1:42. doi:10.1186/s40814-015-0039-6

23 Bellg AJ, Resnick B, Minicucci DS, *et al.* Enhancing treatment fidelity in health behavior change studies: Best practices and recommendations from the NIH Behavior Change Consortium. *Health Psychology* 2004;23:443–51. doi:10.1037/0278-6133.23.5.443

24 National Institute for Health and Care Excellence. Behaviour Change at population, community and individual levels. Public Health Guidance 6. London: NIHCE 2007.

25 Craig P, Dieppe P, Macintyre S, *et al.* Developing and evaluating complex interventions: new guidance. London: Medical Research Council 2008. http://www.mrc.ac.uk/Utilities/Documentrecord/index.htm?d=MRC004871 (Accessed 28 Sep 2018)

26 ISRCTN Registry. ISRCTN17993825 Journeying through dementia: exploring the clinical and cost-effectiveness of a group self-management intervention for people in the early stages of dementia. 2019. http://www.isrctn.com/ISRCTN17993825 (accessed 15 Jan 2019)

27 Alzheimer's Disease International. Participation in dementia trials and studies: Challenges and recommendations. 2014. https://www.alz.co.uk/sites/default/files/pdfs/dementia-trials.pdf (accessed 28 Sept 2018)

28 Herschell AD, Kolko DJ, Baumann BL, *et al.* The role of therapist training in the implementation of psychosocial treatments: A review and critique with recommendations. *Clin Psychol Rev* 2010;30:448–66. doi:10.1016/j.cpr.2010.02.005

29 Mountain GA, Craig CL. What should be in a self-management programme for people with early dementia? *Aging Ment Health* 2012;16:576–83. doi:10.1080/13607863.2011.651430

30 Mulhern B, Rowen D, Brazier J, *et al.* Development of DEMQOL-U and DEMQOL-PROXY-U: Generation of preference-based indices from DEMQOL and DEMQOL-PROXY for use in economic evaluation. *Health Technol Assess (Rockv)* 2013;17:1–140. doi:10.3310/hta17050

31 Smith S, Lamping D, Banerjee S, *et al.* Measurement of health-related quality of life for people with dementia: development of a new instrument (DEMQOL) and an evaluation of current methodology. *Health Technol Assess (Rockv)* 2005;9:1–93. doi:https://doi.org/10.3310/hta9100

32 Herdman M, Gudex C, Lloyd A, *et al.* Development and preliminary testing of the new five-level version of EQ-5D (EQ-5D-5L). *Qual Life Res* 2011;20:1727–36. doi:10.1007/s11136-011-9903-x

33 Hounsome N, Orrell M, Edwards RT. EQ-5D as a quality of life measure in people with dementia and their carers: Evidence and key issues. *Value Health* 2011;14:390–9. doi:10.1016/j.jval.2010.08.002

34 Spitzer RL, Williams JBW, Kroenke K. Test Review: Patient Health Questionnaire-9 (PHQ-9). *Rehabil Couns Bull* 2014;57:246–8. doi:10.1177/0034355213515305

35 Kroenke K, Spitzer RL, Williams JBW. The PHQ-9: Validity of a Brief Depression Severity Measure. *J Gen Intern Med* 2001;16:606–13. doi:10.1046/j.1525-1497.2001.016009606.x

36 Löwe B, Decker O, Müller S, *et al.* Validation and standardization of the generalized anxiety

For peer review only

disorder screener (GAD-7) in the general population. *Medical Care* 2008;46:266–74. doi:10.1097/MLR.0b013e318160d093

37 Schwarzer R, Jerusalem M. Generalized Self-Efficacy scale. In: Weinman J, Wright S, Johnston M, eds. Measures in health psychology: A user's portfolio. Causal and control beliefs. Windsor: NFER-NELSON 1995. 35–7.

38 Diener E, Wirtz D, Tov W, *et al.* New measures of well-being: Flourishing and positive and negative feelings. In: Diener E, ed. Social Indicators Research Series. New York: Springer Science + Business Media 2009. 247–66.

39 De Vries R, Ryan KA, Stanczyk A, *et al.* Public's approach to surrogate consent for dementia research: Cautious pragmatism. *Am J Geriatr Psychiatry* 2013;21:364–72. doi:10.1016/j.jagp.2012.11.010

40 Lawton MP, Brody EM. Assessment of Older People : Self-Maintaining and Instrumental Activities of Daily Living 1. *Gerontologist* 1969;9:179–86. doi:10.1093/geront/9.3_Part_1.179

41 Vernooij-Dassen MJFJ, Persoon JMG, Felling AJA. Predictors of sense of competence in caregivers of demented persons. *Soc Sci Med* 1996;43:41–9. doi:10.1016/0277-9536(95)00332-0

42 Jansen APD, van Hout HPJ, van Marwijk HWJ, *et al.* Sense of competence questionnaire among informal caregivers of older adults with dementia symptoms: A psychometric evaluation. *Clin Pract Epidemiol Ment Health* 2007;3:1–11. doi:10.1186/1745-0179-3-11

43 Moniz-Cook E, Vernooij-Dassen M, Woods R, *et al.* A European consensus on outcome measures for psychosocial intervention research in dementia care. *Aging Ment Heal* 2008;1:29. doi:10.1080/13607860801919850

44 Schulz KF, Altman DG, Moher D. CONSORT 2010 Statement : updated guidelines for reporting parallel group randomised trials. *BMJ* 2010;340:332. doi:10.1136/bmj.c332

45 Baldwin SA, Bauer DJ, Stice E, *et al.* Evaluating models for partially clustered designs. *Psychol Methods* 2011;16:149–65. doi:10.1037/a0023464

46 Walters SJ. Therapist effects in randomised controlled trials: What to do about them. *J Clin Nurs* 2010;19:1102–12. doi:10.1111/j.1365-2702.2009.03067.x

47 Roberts C, Batistatou E, Roberts SA. Design and analysis of trials with a partially nested design and a binary outcome measure. *Stat Med* 2015;35:1616–36. doi:10.1002/sim.6828

48 Kahan BC, Morris TP. Improper analysis of trials randomised using stratified blocks or minimisation. *Stat Med* 2012;31:328–40. doi:10.1002/sim.4431

49 NHS Improvement. Reference Costs 2017/18. 2018. https://improvement.nhs.uk/resources/reference-costs/ (accessed 15 Jan 2019)

50 Curtis L, Burns A. Unit Costs of Health & Social Care 2018. Canterbury: University of Kent 2018.

51 Schafer JL. Multiple imputation: A primer. *Stat Methods Med Res* 1999;8:3–15. doi:10.1191/096228099671525676

52 Van Hout BA, Al MJ, Gordon GS, *et al.* Costs, effects and C/E-ratios alongside a clinical trial. *Health Econ* 1994;3:309–19. doi:10.1002/hec.4730030505

For peer review only

53 McHugh ML. Interrater reliability: the kappa statistic. *Biochem Med (Zagreb)* 2012;22:276–82. doi:10.11613/BM.2012.031

54 Sprange K., Mountain GA, Brazier J, *et al.* Lifestyle Matters for maintenance of health and wellbeing in people aged 65 years and over: study protocol for a randomised controlled trial. *Trials* 2013;14:302. doi:10.1186/1745-6215-14-302

55 Ritchie J, Spencer L. Qualitative Data Analysis for Applied Policy Research. In: Humberman AM, Miles MB, eds. The Qualitative Researcher's Companion. London: SAGE Publications 2002. 305–29. doi:10.4135/9781412986274

56 Cooper C, Ketley D, Livingston G. Systematic review and meta-analysis to estimate potential recruitment to dementia intervention studies. *Int J Geriatr Psychiatry* 2014;29:515–25. doi:10.1002/gps.4034

57 HRA. GDPR guidance for researchers and study coordinators. London: Health Research Authority (HRA) 2019. https://www.hra.nhs.uk/planning-and-improving-research/policies-standards-legislation/data-protection-and-information-governance/gdpr-guidance/ (accessed 18 Jun 2019)

FIGURE LEGENDS

Figure 1: Participant flow through the study

For peer review only

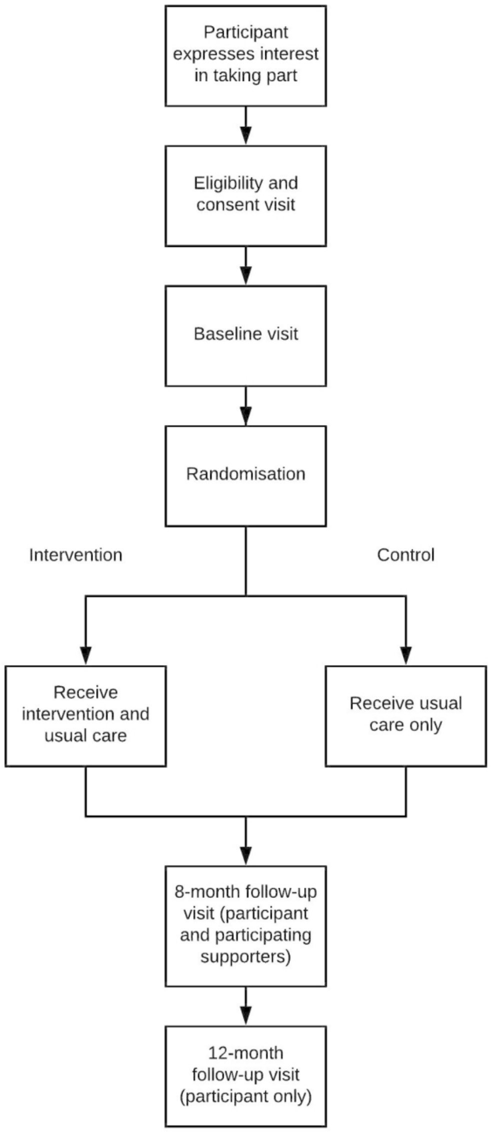

Figure 1 Participant flow through the study

92x190mm (300 x 300 DPI)

STANDARD PROTOCOL ITEMS: RECOMMENDATIONS FOR INTERVENTIONAL TRIALS

SPIRIT 2013 Checklist: Recommended items to address in a clinical trial protocol and related documents*

Section/item	Item No	Description	Addressed on page number
**Administrative information**			
Title	1	Descriptive title identifying the study design, population, interventions, and, if applicable, trial acronym	i
Trial registration	2a	Trial identifier and registry name. If not yet registered, name of intended registry	2
	2b	All items from the World Health Organization Trial Registration Data Set	See 2a
Protocol version	3	Date and version identifier	14
Funding	4	Sources and types of financial, material, and other support	14
Roles and responsibilities	5a	Names, affiliations, and roles of protocol contributors	i-ii
	5b	Name and contact information for the trial sponsor	13
	5c	Role of study sponsor and funders, if any, in study design; collection, management, analysis, and interpretation of data; writing of the report; and the decision to submit the report for publication, including whether they will have ultimate authority over any of these activities	n/a
	5d	Composition, roles, and responsibilities of the coordinating centre, steering committee, endpoint adjudication committee, data management team, and other individuals or groups overseeing the trial, if applicable (see Item 21a for data monitoring committee)	13

For peer review only

Introduction

Background and rationale	6a	Description of research question and justification for undertaking the trial, including summary of relevant studies (published and unpublished) examining benefits and harms for each intervention	____2-3________
	6b	Explanation for choice of comparators	____2-3________
Objectives	7	Specific objectives or hypotheses	____3_________
Trial design	8	Description of trial design including type of trial (eg, parallel group, crossover, factorial, single group), allocation ratio, and framework (eg, superiority, equivalence, noninferiority, exploratory)	____3-4________

Methods: Participants, interventions, and outcomes

Study setting	9	Description of study settings (eg, community clinic, academic hospital) and list of countries where data will be collected. Reference to where list of study sites can be obtained	______5_______
Eligibility criteria	10	Inclusion and exclusion criteria for participants. If applicable, eligibility criteria for study centres and individuals who will perform the interventions (eg, surgeons, psychotherapists)	____4-5________
Interventions	11a	Interventions for each group with sufficient detail to allow replication, including how and when they will be administered	____5-6____
	11b	Criteria for discontinuing or modifying allocated interventions for a given trial participant (eg, drug dose change in response to harms, participant request, or improving/worsening disease)	________n/a_____
	11c	Strategies to improve adherence to intervention protocols, and any procedures for monitoring adherence (eg, drug tablet return, laboratory tests)	______8-9_____
	11d	Relevant concomitant care and interventions that are permitted or prohibited during the trial	______6_____
Outcomes	12	Primary, secondary, and other outcomes, including the specific measurement variable (eg, systolic blood pressure), analysis metric (eg, change from baseline, final value, time to event), method of aggregation (eg, median, proportion), and time point for each outcome. Explanation of the clinical relevance of chosen efficacy and harm outcomes is strongly recommended	_____6-7__
Participant timeline	13	Time schedule of enrolment, interventions (including any run-ins and washouts), assessments, and visits for participants. A schematic diagram is highly recommended (see Figure)	____Table 1 and figure 1_____

For peer review only - http://bmjopen.bmj.com/site/about/guidelines.xhtml

Sample size	14	Estimated number of participants needed to achieve study objectives and how it was determined, including clinical and statistical assumptions supporting any sample size calculations	___9______
Recruitment	15	Strategies for achieving adequate participant enrolment to reach target sample size	_____5_______

Methods: Assignment of interventions (for controlled trials)

Allocation:

Sequence generation	16a	Method of generating the allocation sequence (eg, computer-generated random numbers), and list of any factors for stratification. To reduce predictability of a random sequence, details of any planned restriction (eg, blocking) should be provided in a separate document that is unavailable to those who enrol participants or assign interventions	_____4________
Allocation concealment mechanism	16b	Mechanism of implementing the allocation sequence (eg, central telephone; sequentially numbered, opaque, sealed envelopes), describing any steps to conceal the sequence until interventions are assigned	____4_________
Implementation	16c	Who will generate the allocation sequence, who will enrol participants, and who will assign participants to interventions	____4_______
Blinding (masking)	17a	Who will be blinded after assignment to interventions (eg, trial participants, care providers, outcome assessors, data analysts), and how	___4______
	17b	If blinded, circumstances under which unblinding is permissible, and procedure for revealing a participant's allocated intervention during the trial	______4_______

Methods: Data collection, management, and analysis

Data collection methods	18a	Plans for assessment and collection of outcome, baseline, and other trial data, including any related processes to promote data quality (eg, duplicate measurements, training of assessors) and a description of study instruments (eg, questionnaires, laboratory tests) along with their reliability and validity, if known. Reference to where data collection forms can be found, if not in the protocol	____6-8_______
	18b	Plans to promote participant retention and complete follow-up, including list of any outcome data to be collected for participants who discontinue or deviate from intervention protocols	____6-7_______

Data management	19	Plans for data entry, coding, security, and storage, including any related processes to promote data quality (eg, double data entry; range checks for data values). Reference to where details of data management procedures can be found, if not in the protocol	__12
Statistical methods	20a	Statistical methods for analysing primary and secondary outcomes. Reference to where other details of the statistical analysis plan can be found, if not in the protocol	____9-11_
	20b	Methods for any additional analyses (eg, subgroup and adjusted analyses)	__11_
	20c	Definition of analysis population relating to protocol non-adherence (eg, as randomised analysis), and any statistical methods to handle missing data (eg, multiple imputation)	_10-11___
**Methods: Monitoring**			
Data monitoring	21a	Composition of data monitoring committee (DMC); summary of its role and reporting structure; statement of whether it is independent from the sponsor and competing interests; and reference to where further details about its charter can be found, if not in the protocol. Alternatively, an explanation of why a DMC is not needed	____13_____
	21b	Description of any interim analyses and stopping guidelines, including who will have access to these interim results and make the final decision to terminate the trial	______n/a______
Harms	22	Plans for collecting, assessing, reporting, and managing solicited and spontaneously reported adverse events and other unintended effects of trial interventions or trial conduct	___12-13____
Auditing	23	Frequency and procedures for auditing trial conduct, if any, and whether the process will be independent from investigators and the sponsor	____12______
**Ethics and dissemination**			
Research ethics approval	24	Plans for seeking research ethics committee/institutional review board (REC/IRB) approval	______14_______
Protocol amendments	25	Plans for communicating important protocol modifications (eg, changes to eligibility criteria, outcomes, analyses) to relevant parties (eg, investigators, REC/IRBs, trial participants, trial registries, journals, regulators)	_______13______

Consent or assent	26a	Who will obtain informed consent or assent from potential trial participants or authorised surrogates, and how (see Item 32)	______5______
	26b	Additional consent provisions for collection and use of participant data and biological specimens in ancillary studies, if applicable	__See supplementary information__
Confidentiality	27	How personal information about potential and enrolled participants will be collected, shared, and maintained in order to protect confidentiality before, during, and after the trial	___12-13_____
Declaration of interests	28	Financial and other competing interests for principal investigators for the overall trial and each study site	_____14______
Access to data	29	Statement of who will have access to the final trial dataset, and disclosure of contractual agreements that limit such access for investigators	_____12____
Ancillary and post-trial care	30	Provisions, if any, for ancillary and post-trial care, and for compensation to those who suffer harm from trial participation	_____n/a______
Dissemination policy	31a	Plans for investigators and sponsor to communicate trial results to participants, healthcare professionals, the public, and other relevant groups (eg, via publication, reporting in results databases, or other data sharing arrangements), including any publication restrictions	_____13___
	31b	Authorship eligibility guidelines and any intended use of professional writers	______n/a______
	31c	Plans, if any, for granting public access to the full protocol, participant-level dataset, and statistical code	______12______
**Appendices**			
Informed consent materials	32	Model consent form and other related documentation given to participants and authorised surrogates	See supplementary materials_____
Biological specimens	33	Plans for collection, laboratory evaluation, and storage of biological specimens for genetic or molecular analysis in the current trial and for future use in ancillary studies, if applicable	_____n/a_______ _

For peer review only - http://bmjopen.bmj.com/site/about/guidelines.xhtml

*It is strongly recommended that this checklist be read in conjunction with the SPIRIT 2013 Explanation & Elaboration for important clarification on the items. Amendments to the protocol should be tracked and dated. The SPIRIT checklist is copyrighted by the SPIRIT Group under the Creative Commons "Attribution-NonCommercial-NoDerivs 3.0 Unported" license.

For peer review only

BMJ Open

A study protocol for a randomised controlled trial assessing the clinical and cost-effectiveness of the Journeying through Dementia (JtD) intervention compared to usual care

Journal:	*BMJ Open*
Manuscript ID	bmjopen-2019-029207.R2
Article Type:	Protocol
Date Submitted by the Author:	09-Aug-2019

Complete List of Authors:	Wright, Jessica; University of Sheffield, Clinical Trials Research Unit<br>Foster, Alexis; University of Sheffield, School of Health and Related Research<br>Cooper, Cindy; The University of Sheffield, Sheffield Clinical Trials Research Unit, School of Health and Related Research<br>Sprange, Kirsty; The University of Nottingham, Nottingham Clinical Trials Research Unit<br>Walters, Stephen; University of Sheffield, ScHARR<br>Berry, Katherine; The University of Manchester, School of Psychological Sciences<br>Moniz-Cook , Esme ; The University of Hull, School of Health and Social Work<br>Loban, Amanda; University of Sheffield, School of Health and Related Research<br>Young, Tracey; The University of Sheffield, School of Health and Related Research<br>Craig, Claire; Sheffield Hallam University<br>Dening, Tom; University of Nottingham, Division of Psychiatry & Applied Psychology, School of Medicine<br>Lee, Ellen; University of Sheffield, Clinical Trials Research Unit<br>Beresford-Dent, Julie; University of Bradford, Centre for Applied Dementia Studies<br>Thompson, Benjamin; The University of Sheffield, School of Health and Related Research<br>Young, Emma; The University of Sheffield, School of Health and Related Research<br>Thomas, Benjamin; The University of Sheffield, School of Health and Related Research<br>Mountain, Gail; University of Bradford, Centre for Applied Dementia Studies, Faculty of Health Studies

**Primary Subject Heading**:	Geriatric medicine
Secondary Subject Heading:	Mental health, Health economics, Qualitative research
Keywords:	Randomised Controlled Trial, Dementia < NEUROLOGY, Research Protocol, Post diagnostic support, Self-management, Well-being

TITLE PAGE

Title

A study protocol for a randomised controlled trial assessing the clinical and cost-effectiveness of the Journeying through Dementia (JtD) intervention compared to usual care

Corresponding author

Jessica Wright
Journeying Through Dementia (JtD) Trial,
Sheffield Clinical Trials Research Unit,
ScHARR, The University of Sheffield,
Regent's Court,
30 Regent St, Sheffield S1 4DA
Telephone: 0114 222 4304
Email: Jessica.wright@sheffield.ac.uk

Author Names/Affiliations

Jessica Wright, Sheffield Clinical Trials Research Unit, School of Health and Related Research, The University of Sheffield, Sheffield, UK

Alexis Foster, School of Health and Related Research, The University of Sheffield, Sheffield, UK

Cindy Cooper, Sheffield Clinical Trials Research Unit, School of Health and Related Research, The University of Sheffield, Sheffield, UK

Kirsty Sprange, Nottingham Clinical Trials Research Unit, The University of Nottingham, Nottingham, UK

Stephen Walters, School of Health and Related Research, The University of Sheffield, Sheffield, UK

Katherine Berry, Manchester Institute for Collaborative Research on Ageing, The University of Manchester, Manchester, UK

Esme Moniz-Cook, School of Health and Social Work, The University of Hull, Hull, UK

Amanda Loban, Sheffield Clinical Trials Research Unit, School of Health and Related Research, The University of Sheffield, Sheffield, UK

Tracey Young, School of Health and Related Research, The University of Sheffield, Sheffield, UK

Claire Craig, Art & Design Research Centre, Sheffield Hallam University, Sheffield, UK

Tom Dening, Division of Psychiatry & Applied Psychology, School of Medicine, University of Nottingham, Nottingham, UK

Ellen Lee, Sheffield Clinical Trials Research Unit, School of Health and Related Research, The University of Sheffield, Sheffield, UK

For peer review only - http://bmjopen.bmj.com/site/about/guidelines.xhtml

Julie Beresford-Dent, Centre for Applied Dementia Studies, University of Bradford, Bradford, UK

Benjamin John Thompson, Sheffield Clinical Trials Research Unit, School of Health and Related Research, The University of Sheffield, Sheffield, UK

Emma Louise Young, Sheffield Clinical Trials Research Unit, School of Health and Related Research, The University of Sheffield, Sheffield, UK

Benjamin David Thomas, Sheffield Clinical Trials Research Unit, School of Health and Related Research, The University of Sheffield, Sheffield, UK

Gail Mountain, Centre for Applied Dementia Studies, Faculty of Health Studies, University of Bradford, Bradford, UK

Word Count: 6648 (excluding references, abstract, tables and figures)

For peer review only

For peer review only - http://bmjopen.bmj.com/site/about/guidelines.xhtml

ABSTRACT

Introduction

Services are being encouraged to provide post diagnostic treatment to those with dementia but the availability of evidence-based interventions following diagnosis has not kept pace with increase in demand. To address this need, the Journeying through Dementia (JtD) intervention was created. A randomised controlled trial (RCT), based on a pilot study, is in progress.

Methods and analysis

The RCT is a pragmatic, two-arm, parallel group trial designed to test the clinical and cost-effectiveness of JtD compared to usual care. Recruitment will be through NHS services, third sector organisations and Join Dementia Research. The sample size is 486 randomised (243 to usual care and 243 to the intervention usual care). Participants can choose to ask a friend or relative (supporter) to become involved in the study. The primary outcome measure for participants is Dementia Related Quality of Life (DEMQOL), collected at baseline and at 8 months' post randomisation. Secondary outcome measures will be collected from participants and supporters at those visits. Participants will also be followed-up at 12 months' post randomisation with a reduced set of measures. A process evaluation will be conducted through qualitative and fidelity sub-studies.

Analyses will compare the two arms of the trial on an intention to treat as allocated basis. The primary analyses will compare the mean DEMQOL scores of the participants at 8 months between the two study arms. A cost-effectiveness analysis will consider the incremental cost per Quality Adjusted Life Years of the intervention compared with usual care. Qualitative and fidelity sub-studies will be analysed through framework analysis and fidelity assessment tools respectively.

Ethics and dissemination

REC and HRA approval were obtained. A Data Monitoring and Ethics Committee has been constituted. Dissemination will be via publications, conferences and social media. Intervention materials will be made open access.

Trial registration number

ISRCTN17993825

For peer review only

STRENGTHS AND LIMITATIONS OF THE STUDY

- People living with dementia were involved in developing the content of the Journeying through Dementia (JtD) intervention and are involved in advising the study.
- The JtD intervention includes sessions without supporters present, to help develop independence and confidence, and is one of the only interventions that people with dementia can participate in without supporters.
- The JtD study will recruit up to 500 participants and is therefore one of the largest trials of a psychosocial intervention for people with dementia in the UK.
- The potential for unblinding of researchers when arranging or attending follow-up visits is a limitation but this will be monitored and minimized by not sending unblind researchers to visits.
- Recruitment is known to be challenging in this population but we plan to try multiple pathways for recruitment including through services, the Join Dementia Research database, promotion and the third sector.

INTRODUCTION

The impact on the economy, for services and for individuals living with dementia and their family carers is larger for dementia than for all other long-term illnesses in people aged 60 and over.[1] Two thirds of people with dementia live in the community, and half of these require some form of support.[2] As a result, dementia research (both for cure and for care) in the NHS and social care is important, and this is reflected in UK health policy.[3] In 2009, the UK Government announced a National Dementia Strategy, which mandated the establishment of memory services and aimed to increase the rates of early diagnosis and improve support for people in the early stages of dementia.[4]

The National Audit of Memory Services (2013) found there had been a fourfold increase in numbers presenting since 2010/11, and in 2013 49.3% were in the early stages of the condition.[5] Earlier diagnosis allows individuals to receive treatment earlier and enables the individual and memory services to plan more effectively for the future.[6] Memory services have been strongly encouraged to provide post diagnostic treatment and support,[7,8] but the availability of appropriate evidence-based interventions has not kept pace with the increase in demand, in particular for those with early stages of dementia. The lack of appropriate interventions has led to inconsistency between Trusts regarding what is being offered to people post diagnosis.

The potential value of psychosocial interventions for people in the early stages of dementia is recognised[9–12] and is also driven by the knowledge that a cure for dementia is unlikely in the near future. Psychosocial interventions are diverse but a common theme is that they do not involve the use of medication and instead focus on supporting people to overcome challenges and maintain independence and well-being. However, whilst there has been some shift, the use of psychosocial interventions within dementia care has been a neglected area for both research and practice.[11]

There is a growing body of evidence to demonstrate how individuals with dementia can be supported to use self-management based techniques (sometimes in combination with other interventions such as cognitive rehabilitation and occupational therapy).[13–18] A qualitative study

For peer review only - http://bmjopen.bmj.com/site/about/guidelines.xhtml

of people with dementia who attended a self-management programme reported that participants identified the opportunity for peer support as being beneficial and considered that the programme could be improved by greater emphasis being placed upon maintaining activities and relationships and improving positive wellbeing.[19] The Healthbridge evaluation[20] and the Mental Health Foundation evaluation[21] found evidence that people with dementia and their carers can benefit from receiving group based peer support.

The Journeying through Dementia (JtD) intervention was developed from the Lifestyle Matters programme and a pilot study was conducted to examine the feasibility of a future population-based larger trial of this intervention.[22] The intervention was found to be acceptable to both people with dementia and their carers. Reported benefits included increased confidence and self-efficacy, engagement in activities and re-engagement with fun and friendships.[22] The intervention is manualised, based on occupational therapy principles, and is designed to support independence and well-being. The intervention incorporates elements of self-management and group-based peer support and has been designed to improve the quality of life for people in the early stages of dementia.

The JtD randomised controlled trial (RCT) will test the clinical and cost effectiveness of the JtD intervention. Funding was obtained through the National Institute of Health Research (NIHR) Health Technology Assessment (HTA) theme to conduct the RCT. This paper describes the research protocol for undertaking the RCT which started recruitment in November 2016.

AIMS AND OBJECTIVES

The primary aim of the JtD trial is to determine the clinical and cost-effectiveness of the JtD intervention for people in the early stages of dementia. To meet this aim, the objectives are to:

1. Conduct an internal pilot RCT of the intervention to check the feasibility of rates of recruitment at scale.
2. Proceed to a full a pragmatic RCT evaluating the clinical and cost-effectiveness of the JtD intervention.
3. Conduct fidelity checks regarding the delivery of the JtD intervention.
4. Undertake an embedded qualitative sub-study to explore issues concerned with intervention delivery.
5. Identify how the intervention might be realistically delivered through services.

METHODS

Trial design

The JtD trial is a pragmatic, two-arm, parallel group, individually randomised RCT. It uses a superiority framework to deliver an intention-to-treat comparison of the JtD intervention with usual care.

The trial includes three sub-studies. The first is a health economics evaluation using a cost-effectiveness analysis of the incremental cost per Quality Adjusted Life Year (QALY) of the JtD intervention compared with usual care.

For peer review only - http://bmjopen.bmj.com/site/about/guidelines.xhtml

For peer review only

The second sub-study is a fidelity assessment as part of the process evaluation, using an intervention fidelity framework based on that identified by the Behaviour Change Consortium and National Institute for Health and Care Excellence (NICE).[23,24]

The third sub-study is a qualitative study, part of the process evaluation, in line with Medical Research Council (MRC) guidance on developing and evaluating complex interventions.[25] The qualitative sub-study will involve semi-structured interviews with participants, supporters, facilitators and supervisors.

Randomisation, blinding and bias

To minimise bias, allocation will be concealed through the use of a centralised web-based randomisation service. The randomisation sequence will be stratified by delivery site and constrained by a fixed block size to ensure participants are allocated evenly to each arm of the trial at each delivery site. Participants will be randomised in equal numbers to intervention and control arms. An unblind member of the research team who will not be conducting outcome assessments will enter the participants' details into the randomisation system and inform the relevant parties of the outcome.

Members of the Trial Steering Committee (TSC), study statisticians, health economists and outcome assessors will be blinded to treatment allocation whilst the trial is ongoing. For practical reasons linked to the provision of a centralised web-based randomisation service and the set-up and delivery of the intervention groups, some members of the research team will not be blinded, including the Trial Manager and Chief Investigator. Due to the nature of the intervention, participants will not be blinded. If the outcome assessors know (or suspect) they have been unblinded this will be recorded on an unblinding form.

We protect against facilitator bias where the same facilitators also provide usual care in two ways. The first is that usual care is limited and often restricted to NICE recommended cognitive stimulation therapy which would not be readily influenced by training in delivery of JtD, as it follows a detailed and prescriptive session-by-session plan of group exercises which are facilitator-led. Other less common post-diagnostic services include Living Well with Dementia or memory groups, which do contain some features of JtD, but not enactment of learnt skills in the community or the mix of individual and group sessions JtD incorporates. The second way is that a proportion of the facilitators recruited will not deliver usual care, as approximately 25% of facilitators are trained research staff. A further form of bias, that caused by cross-contamination of participants between the two study arms, is considered as unlikely as post diagnostic services for people living with dementia are limited and cognitive stimulation therapy is more likely to be offered later in the dementia trajectory. Extended post diagnostic follow-up is not common so it is unlikely participants from different study arms would meet at routine appointments and discuss involvement.

Participants

Persons with early-stage dementia will be approached to take part in the trial. A family member, friend or neighbour that provides support to the study participant (referred to as the 'supporter') can be approached to take part in the trial, but only if invited to do so by the person with dementia.

For peer review only - http://bmjopen.bmj.com/site/about/guidelines.xhtml

The study participant can also choose not to invite a supporter to take part in the trial and still take part. Participating supporters will automatically be randomised to the same arm as the participant. See Figure 1 for participant flow through the study.

(Insert Figure 1 here)

Eligibility criteria

Participants will be eligible for the study if they:

1. have a diagnosis of any form of dementia,
2. have a Mini Mental State Examination (MMSE) score of 18 or more, measured less than 2 months' pre-consent,
3. have capacity to make informed decisions,
4. are living in the community in their own or sheltered accommodation (those living in residential or nursing care are not eligible),
5. are willing to attend the JtD intervention,
6. are able to converse and communicate in English, and
7. are not taking part in any other pharmacological or psychosocial intervention studies at the time of enrolment.

Supporters will be eligible to participate if they are aged 18 years or over, are named by the person with dementia as their supporter, the person with dementia wishes them to take part, they can converse and communicate in English, and have the ability to give informed consent.

Site selection and participant recruitment

The JtD trial will operate in England within 13 NHS trusts with specialist dementia services. The sites will be selected based on a convenience sample of locations clustered geographically around the north and midlands (for a list of sites, see study ISRCTN webpage[26]).

Previous studies involving persons with dementia have shown that recruiting participants to such studies can be challenging.[27] To ensure sufficient numbers are recruited to the study a number of recruitment pathways will be used: referral from clinical teams; mail-outs to eligible patients via primary or secondary care clinical teams; study promotion via posters etc.; third sector organisations; and the Join Dementia Research database. The research team will contact interested participants and send further information about the study. Clinical teams will check with the potential participants that they are willing to be approached by the research team before an approach is made.

To enable potential participants to make a direct approach to the research team, a reply card will be designed which can be completed, sealed and returned to the central research team who will then pass the information on to relevant local site researchers. These reply cards will be distributed with information sheets at events including dementia cafes or posted as part of mail-outs to GPs. Local promotions, for example via posters in clinics or GPs, will include the telephone and email details of local researchers for direct contact to take place.

For peer review only - http://bmjopen.bmj.com/site/about/guidelines.xhtml

A consent and eligibility visit will be arranged if an individual would like to take part where trained researchers will assess eligibility and request written informed consent.

Intervention

JtD is a manualised intervention consisting of twelve weekly facilitated groups with 8-12 participants with dementia, which take place over successive weeks. Each participant also receives four one-to-one sessions with one of the intervention facilitators, to pursue individual goals. The first one-to-one session takes place before the start of the group intervention, with the remaining three being scheduled during the 12 weeks and at locations and times agreed with the participant. The group aspect of intervention should be delivered in a community venue.

The content of the JtD intervention involves:

a. Ways of thinking about dementia (what is dementia, effects on everyday life, challenging stereotypes, sharing coping strategies).
b. Keeping physically well (relationship between physical and mental wellbeing, embedding health activity in everyday life, diet).
c. Memory (strategies to aid memory, impact on everyday life and learning and practicing new techniques).
d. Keeping mentally well (relationships between anxiety and memory, and dementia and stress).
e. Endings (celebration of achievements and how to move forward).

Participants are encouraged to select different topics from the manualised intervention and explore them with guidance and suggestions from facilitators. Participants are also able to suggest topics not within the manual. One essential component of the intervention is the enactment of activities, particularly in the community; three of the twelve group meetings should be 'out of venue' activities. Participants are able to invite a supporter to participate in the group aspect of the intervention during sessions 1, 6 and 12, and in the individual sessions if the participant finds this helpful in achieving their goals.

The intervention should be facilitated by a minimum of two NHS staff who are experienced with working with people with dementia. Facilitators will normally be someone employed on Agenda for Change Bands 3-5. Facilitators must receive a two-day training course prior to delivering the intervention. In some cases, for example if they are a reserve facilitator, they may receive a shortened course supported by online resources created for this purpose. Facilitators will be supervised by a colleague experienced in supervision who has also attended the facilitator training and is of suitable seniority and experience. The supervisors themselves will be supervised by a member of the central study team who is a Clinical Psychologist and is experienced in the 'train the trainer' method[28].

Patient and public involvement (PPI)

People living with dementia were involved in developing the content of JtD[29] and in the feasibility study.[22] The JtD Trial Steering Committee will include a member who is living with dementia. Additionally, an advisory group of people living with dementia (experts by experience) will meet at

For peer review only - http://bmjopen.bmj.com/site/about/guidelines.xhtml

intervals throughout to provide input into study materials. We also plan to involve people living with dementia in some aspects of the qualitative data analysis and in creating and delivering the study dissemination plans.

OUTCOMES

Outcomes were identified through a feasibility study which identified appropriate measures and tested their application.[22] The burden of questionnaires was found to be acceptable.

The primary outcome is the Dementia Related Quality of Life (DEMQOL)[30,31] measure at 8 months post randomisation.

The secondary outcomes are:

- European Quality of Life - 5 Dimensions (EQ-5D-5L)[32,33]
- Patient Health Questionnaire-9 (PHQ-9)[34,35]
- Generalised Anxiety Disorder-7 (GAD-7)[36]
- General Self-Efficacy Scale (GSE)[37]
- Diener's Flourishing Scale (DFS)[38]
- Self-Management Ability Scale (SMAS)[39]
- Instrumental Activities of Daily Living (IADL)[40]
- Health and Social Care Resource Use Questionnaire (HSCRU)[22]
- Sense of Competency Questionnaire (SCQ)[41],[42]

See Table 1 (below) for further information on who completes which measures in the study. The outcome measures used were selected to measure the following key components of the intervention: mental wellbeing or mood (DEMQOL, PHQ-9, GAD-7); building relationships and a sense of connectedness (DFS, SMAS); self-management (SMAS); belief that life is meaningful despite dementia (DEMQOL, GSE, DFS, SMAS); and Instrumental Activities of Daily Living (IADL) and strategies to maintain cognitive functioning (IADL). Additionally, they also support analysis of the cost-effectiveness of the intervention (DEMQOL, EQ-5D-5L, HSCRU) and the participating supporters' perceptions of competence (SCQ). Dementia specific outcome measures are those recommended for use across Europe and are selected for self rather than proxy completion.[43] Non dementia specific outcome measures were selected if there was no appropriate dementia specific measure available.

DATA COLLECTION

Quantitative

Data will be collected from all participants living with dementia at eligibility/consent, baseline, 8 and 12 months post randomisation and from consented participating supporters at baseline and 8 months' post randomisation (see Table 1). There is a reduced set of measures linked to the 12 month visit as we require limited further information on quality of life and health and social care resource use at that time-point; the key outcome point is at 8 months. The outcome measures will be interviewer-administered at face-to-face visits by blinded outcome assessors who have received training to deliver the measures to people living with dementia and their supporters. The follow-up outcome measures will be collected 2 weeks pre and 8 weeks post the date they are due. Participant

For peer review only - http://bmjopen.bmj.com/site/about/guidelines.xhtml

retention will be promoted by regular communication with the participants and supporters through communication including newsletters and Christmas cards.

Table 1 Outcome measures and time-points for collection. * Denotes the primary outcome measure.

Measure	Participant				Participating Supporter	
	**Eligibility and Consent visit**	**Baseline** <2 months prior to the intervention start date	**8 Months** Due < 2 weeks pre and < 8 weeks post randomisation	**12 Months** Due < 2 weeks pre and < 8 weeks post randomisation if within study timelines	**Baseline** Due <2 months prior to the intervention	**8 Months** Due < 2 weeks pre and < 8 weeks post randomisation
Capacity assessment	✓					
Mini Mental State Examination	✓					
Eligibility checklist	✓				✓	
Baseline demographics	✓				✓	
DEMQOL		✓	✓*	✓		
EQ-5D-5L		✓	✓	✓	✓	✓
PHQ-9		✓	✓		✓	✓
GAD-7		✓	✓			
GSE		✓	✓			
DFS		✓	✓			
SMAS		✓	✓			
IADL		✓	✓	✓		
HSCRU			✓	✓		
SCQ					✓	✓

Visits to collect outcome data will be arranged with the participant by a researcher, in some cases a participating supporter will assist with these arrangements. All visits will be conducted at a time and location most suitable for the participant. When we conduct the follow-ups we will prioritise the importance of the measures in case the participant tires. We will offer a second visit if the participant is tired or otherwise unable to complete the assessments, which will be organised as soon as possible after the first. Participating supporters may not have the time or capacity to receive a face-to-face visit and follow-up outcome measures may therefore be collected from them over the telephone. Similarly, if a participant does not want a visit, a reduced set of outcome measures may be taken by telephone (prioritising collection of DEMQOL and telephone versions of HSCRU and EQ-5D-5L).

Intervention attendance

Records will be kept of all attendances for each participant randomised to the intervention.

Intervention dropout and study withdrawal

For peer review only - http://bmjopen.bmj.com/site/about/guidelines.xhtml

If a participant decides to withdraw either from the intervention or the study, this will be recorded. If the participant just withdraws from the intervention, they will be followed-up unless they explicitly also withdraw consent for follow-up meetings for collection of outcomes (data up to this time will be included in the trial). If the participant fully withdraws from the study no further data will be collected.

Intervention costings

Information on the cost of facilitated group and individual sessions will be collected including hire of local community venues, facilitator salaries and travel, refreshments, and other costs such as administration and materials used.

SAMPLE SIZE

The primary outcome for the study is the mean DEMQOL score 8 months post randomisation. Assuming a standard deviation of 11 points for the DEMQOL, a mean difference of 4 or more points is clinically and practically important.[30] The sample size has been calculated to have a 90% power of detecting this 4 point difference (equivalent to a standardised effect size of 0.36) in group mean scores at eight months as being statistically significant at 5% (two sided) level. As the JtD intervention is a facilitator led intervention with a group component, the outcomes of the participants in the same group with the same facilitators may be clustered. With no adjustment for clustering by facilitator the target sample size would be 160 per arm with a total sample size of 320. We have assumed an average cluster size of 8 people with dementia per facilitated group and an intra-cluster correlation of 0.03; this will inflate the sample size by a design effect of 1.21; to 194 per group (388 total sample size) with valid primary outcome data. Assuming at least a 20% loss to follow-up the target sample size for the trial is to randomise to 243 participants in each arm (n=486).

DATA ANALYSIS

Statistical data analysis

As JtD is a pragmatic parallel group randomised trial, with a usual care (control) arm, data will be reported and presented according to a revised CONSORT statement.[44] Statistical analysis will be performed on an intention-to-treat-basis. All exploratory tests will be two-tailed with alpha = 0.05. Baseline demographics and quality of life data will be described and summarised overall and by treatment group.

The primary analysis will compare mean patient reported DEMQOL scores at 8 months post randomisation between the intervention (JtD) arm and control arms using a mixed effects linear regression model adjusted for DEMQOL baseline score and site and allowing for the clustering of the outcome by the JtD intervention.[45–47] The trial is a partially nested design with comparison of a group therapy (JtD) with individual therapy with clustering in one (intervention) arm. Each person with dementia in the control group (unclustered arm) will be treated as a cluster (singleton) of size one. The cluster indicator will be treated as a random effect. A stratification variable used for randomisation (site) will be included as a fixed factor.[48] A partially clustered mixed effects linear regression model with homoscedastic errors as well as a heteroscedasticity mixed effects linear regression model will also be considered to account for potential differential variability of outcomes between the two treatment groups. A 95% confidence interval (CI) for the mean difference in

For peer review only - http://bmjopen.bmj.com/site/about/guidelines.xhtml

DEMQOL scores between the intervention and control groups will be calculated together with the associated P-value. A further adjusted analysis may also be performed depending on the observed degree of imbalance in baseline covariates (which are of potential prognostic importance) again using a mixed effects linear regression model. Additional covariates (of potential prognostic importance) include other baseline variables, such as age, gender, PHQ-9, and GAD-7. In the event that there are more than 10 couples (20 participants) living under the same roof from different households in the study, then the primary and secondary analyses will be changed to take into account the hierarchical or clustered nature of the data. A multi level mixed effects model will be used; the random effects will be JtD intervention groups (top level) and couple/singles (lower level). Individual participants who are not part of a couple will be treated as clusters of size one.

Participants will be followed up for up to 12 months post randomisation. Mean DEMQOL scores at 12 months follow-up will be compared as described for the primary outcome above.

For the primary outcome, the DEMQOL score at 8 months follow-up, missing data will be imputed through a variety of methods including: regression and multiple imputation as part of a sensitivity analysis.

We will complement the intention to treat (ITT) analysis of the primary outcome with a complier average causal effects analysis (CACE) as a secondary analysis alongside the primary ITT analysis. Compliance will be defined as a binary variable with participants who attend at least 10 of the 16 JtD sessions (both individual and group sessions combined) regarded as being compliant.

There are no planned interim statistical analyses or formal stopping rules in relation to efficacy.

In terms of missing data, the primary analysis will be performed based on participants with available 8 month primary outcome data. Sensitivity analysis on the primary outcome will include multiple imputation using chained equations and regression imputation.

Secondary outcome measures

Secondary outcomes at 8 and 12 months post-randomisation will be compared between the intervention and control groups using a mixed effects linear regression model as for the primary outcome. A 95% CI for the mean difference in this parameter between the treatment groups will also be calculated together with the associated P-value.

Outcome measures for the participating supporters at 8 months will be compared between the intervention and control groups using a mixed effects linear regression model. The mean difference in outcome with associated 95% CI and P-value will be presented for: a) the baseline (specific to the secondary outcome) and site adjusted analysis and b) adjusted analysis with additional covariates in addition to a).

Subgroup analysis

A subgroup analysis using a mixed effect linear regression model, with the primary outcome (DEMQOL) at 8 months post randomisation as the response will be carried out. We will use an interaction statistical test between the randomised intervention group and subgroup to directly examine the strength of evidence for the treatment difference between the treatment groups

For peer review only - http://bmjopen.bmj.com/site/about/guidelines.xhtml

varying between subgroups. Supporter involvement (yes or no) will be the only a priori defined sub groups to be considered for interaction test.

HEALTH ECONOMICS EVALUATION

A trial based economic evaluation will be undertaken of an intention-to-treat comparison of the costs and outcomes of the two trial arms. A cost-effectiveness analysis will be undertaken of the incremental cost per Quality Adjusted Life Year (QALYs) of the JtD intervention compared with usual care provided through NHS memory services. QALYs will be calculated using the EQ-5D-5L preference-based index administered at baseline, 8 and 12 months. A sensitivity analysis will be undertaken using utility values from the DEMQOL-U, which can be derived from responses to the DEMQOL questionnaire.[30] The total cost of the intervention will be estimated at the individual participant level and will include the costs of providing the intervention and the subsequent consequences for the use of routine health and social care services. The average cost per attendance will be calculated and this estimate will be applied to the actual number of group and individual sessions that each participant attended.

The use of services by trial participants will be collected in detail using a Health and Social Care Resource Use (HSCRU) questionnaire administered at 8 and 12 months post randomisation. Service use will be costed using the most recent National Reference Cost Data and Unit Costs of Health and Social Care.[49,50] Missing data will be dealt with using multiple imputation for EQ-5D-5L, DEMQOL-U and resource use data.[51] A random effects linear regression model, accounting for clustering, will be fitted, the model will include baseline scores for EQ-5D-5L and baseline costs. The central analysis of mean incremental costs per QALY will be subjected to a full sensitivity analysis of key parameters including the measure used to estimate QALYs and number of participants at the weekly sessions. A full probabilistic sensitivity analysis will be performed to examine the probability of cost-effectiveness of the intervention for the NHS for different levels of costs and QALY gains.[52]

PROCESS EVALUATION

The process evaluation has been designed to: 1) examine the factors that may influence the fidelity of intervention delivery and 2) explore its perceived impact and acceptability from user and provider perspectives. There are two strands to this, the fidelity assessment and qualitative interviews, explained below.

Fidelity assessment

The fidelity of intervention delivery, as well as of the training and supervision received by facilitators will be assessed on the basis of criteria derived from the intervention protocol and manual. Fidelity checks will adhere to a framework based on that identified by the Behaviour Change Consortium[23] and NICE guidance on behaviour change.[24] The fidelity assessment framework (adapted from Bellg et al, 2004[23]) will assess and monitor for the consistency, facilitator training (in terms of standardised delivery of training and skills acquisition for facilitators), intervention delivery (in terms of standardised delivery between intervention groups) and the receipt of the intervention by intervention group participants.

For peer review only

Training delivery and receipt of the facilitator training will be observed and rated by the same two researchers (the lead for fidelity and one other member of the research team) for inter-rater reliability using a bespoke *Training observation checklist*. To assess facilitator adherence to the manualised intervention and participant receipt of the intervention, a purposive selection of group meetings across sites will also be observed using a *Group observation checklist* which is based on the contents of the intervention and the training. Each selected group will be observed on two occasions to identify facilitator drift or changes in participant behavior.

Frequencies will be used to determine the extent to which the training programme received by facilitators maintained fidelity to what was intended. Data will also be analysed to compare intervention delivery between and across sites to check for consistency. Inter-rater reliability between coders will be determined using the Kappa statistic.[53] Similar methods have been used in previous studies.[54]

Qualitative interviews

An embedded qualitative sub-study will explore the mechanisms of the intervention, for example what elements of the intervention appear to support people to improve their self-management and well-being and what promotes good facilitation of the intervention.

Individual qualitative semi-structured interviews will be conducted with a purposive sample of 20 participants (approximately 10%) from the intervention groups observed in the fidelity sub-study and with approximately 12 participating supporters, preferably supporters of participants who are also being interviewed. A participant interview schedule and supporter interview schedule will be developed to cover the following themes:

- Range and nature of issues that influence experiences of taking part in the intervention
- Factors that may influence the effectiveness, adoption and diffusion of this innovation in the future
- Perceived skills and competencies required to facilitate the intervention
- The barriers and facilitators to uptake and continued use
- The effect of the intervention upon living with dementia
- The effect of the intervention upon the experiences of supporting someone with dementia

Semi-structured interviews will also be conducted with approximately 20% of all facilitators and supervisors across the sites upon completion of their delivery of the intervention. The sample will include a range of sites and facilitators with different levels of experience of delivering the intervention. A facilitator interview schedule and supervisor interview schedule will be created to cover the following themes:

- What issues promote the effectiveness of intervention facilitation
- The skills and competencies required to facilitate the programme
- The barriers and facilitators to its uptake and continued use
- Factors that may mediate or moderate the effectiveness of the intervention

Researchers undertaking participant interviews will be trained to use enhanced methods of communication with people with dementia to try to ensure that meaningful discussion takes place.

For peer review only - http://bmjopen.bmj.com/site/about/guidelines.xhtml

Transcripts of interviews will undergo respondent validation.

Qualitative analysis

Framework analysis[55] will be applied to all interview data.[56] For the purposes of reporting, confidentiality will be maintained by using unique participant identifiers and removing identifiable information. A thematic framework will be agreed by two researchers and an index developed for transcript coding. This will follow the five stages of framework analysis.[55] Findings will be used to identify emergent factors that influence the uptake and impact of the intervention as well as explore potential explanations for the quantitative findings.[56] Further analysis will also be undertaken to triangulate the qualitative data (between facilitator/supervisor and participant/supporter interviews) as well as the fidelity and qualitative data in order to look for between source similarities and divergences. Analysis workshops will be held with people living with dementia and their supporters to respond to and help validate the initial qualitative analysis. The workshop outcomes will be used to refine the qualitative analysis.

DATA MANAGEMENT, CONFIDENTIALITY AND SHARING

Sheffield Clinical Trials Research Unit (CTRU) will provide data management services. Data will be entered remotely on to a centralised web-based data capture system (Prospect) by university researchers and authorised staff at participating NHS sites. The Case Report Form captures trial data and has been specifically designed for this trial. Access to Prospect is controlled by usernames and encrypted passwords. Prospect provides a full electronic audit trail, as well as validation features which will be used to monitor study data quality. The identity of participants will be protected by the removal of any identifiable data prior to dissemination of information, and no identifiable data will be transferred to the statistician or health economist. All participating NHS Sites will be subject to data monitoring reviews to check data entry, consent and eligibility, amongst other items. The trial follows the UK Health Research Authority guidance on the General Data Protection Regulation[57] and has implemented a privacy policy and transparency information appropriate to people living with dementia.

Data sharing

JtD trial data will be held and available for five years after the end of the trial (November 2019). JtD Trial Data will not be archived in a repository, instead data will be released on a case-by-case basis. We shall make data available to the scientific community with as few restrictions as feasible. Data access requests will be reviewed and authorised by a sub-committee of the Trial Management Group (TMG) during the trial and by the Sheffield CTRU after the trial has ended. Access requests will be considered against pre-determined criteria and data sharing will only take place if this aligns with the consent provided by JtD participants. Data will be anonymised prior to being shared.

ETHICS, GOVERNANCE AND SAFETY

Ethical issues

For peer review only - http://bmjopen.bmj.com/site/about/guidelines.xhtml

There are two key ethical issues to take into account. The first is the potential need to break confidentiality where there is a risk of harm. We will request consent to contact the participant's or supporter's GP, or other health professional, in situations where researchers are concerned that there might be risk of harm. For example, two outcome measures used on the study may indicate a need to clinically treat anxiety (GAD-7) or depression (PHQ-9). Additionally, concerns regarding participant safety may also be raised at any stage of the study; e.g. observed deterioration in mental or physical state of participants, safeguarding issues, or of a risk to self or others. We will work alongside local Trust procedures to report any risks appropriately. Local site investigators with clinical backgrounds will be asked to provide advice when appropriate. The responsible healthcare professional or relevant PI will be able to recommend the withdrawal of the participant if they feel it is appropriate. We will record all actions taken.

The second is the risk that during the trial, people with dementia may lose the capacity to consent to continuing participation. As part of the consent process, people with dementia will be asked to nominate a person to act as a consultee we may contact in the event that they lose capacity during the trial. The consultee will be independent from the study and can be a relative, friend or medical professional. All researchers will be provided with guidance and local training on identifying and dealing with capacity issues identified before or during 8 and 12 month follow-up visits. If at any point the participant indicates they do not wish to continue to take part in the trial, they will be withdrawn. If the person with dementia loses capacity during the trial, but indicates they wish to remain in the study, the consultee will be asked to make a judgement, based on their existing and pre-existing knowledge of the person with dementia, about whether they would want to continue participation or not. If the consultee advises that the participant would wish to be withdrawn, the researchers will withdraw them from the study. If the consultee advises that the participant would wish to stay taking part, even though they lack capacity, every effort will be made to accommodate this, for example by reducing the number of outcome measures, or using prompts or other means to help the participant complete the measures. We will record information about the activation of the consultee pathway on the trial.

Governance

The trial is coordinated by the Sheffield CTRU on behalf of the Sponsor. The sponsor of the trial is Sheffield Health and Social Care Foundation Trust, Fulwood House, Old Fulwood Road, Sheffield, S10 3TH. The JtD TMG contains project co-applicants, members of the data management team, the Sponsor, Trial Manager and other representatives, and oversees the operation of the trial and enables communication throughout the Trial, for example to disseminate protocol amendments. An independent Trial Steering Committee (TSC), comprised of an independent statistician, PPI representative and a Senior Clinical Research Associate, provides overall supervision of the trial, advises the CI, oversees protocol modifications, monitors the trial's progress and if necessary closes the trial. An independent Data Monitoring and Ethics Committee (DMEC) comprised of two independent statisticians and an Occupational Therapist Clinical Researcher, reviews the trial protocol, monitors patient safety and advises the TSC if they feel the trial should be prematurely closed.

Safety

For peer review only

A Serious Adverse Event (SAE) reporting system will be used on the Trial. Non-serious adverse Events are not anticipated as a consequence of the intervention and will not be monitored. An SAE either:

a) results in death,
b) is life-threatening (subject at immediate risk of death),
c) requires hospitalisation or prolongation of existing hospitalisation,
d) results in persistent or significant disability or incapacity, or
e) is otherwise considered medically significant by the investigator.

All SAEs will be assessed to see if they are related to the intervention or other trial procedures, and if they are the Sponsor and Research Ethics Committee will be immediately informed. SAEs are periodically reported to the trial's DMEC.

Additionally, we consider safety of the researchers to be extremely important and have developed a lone worker policy. The researcher must complete a form detailing information about any participant visits and their contact information and provide this to a 'buddy' who will ensure the safety of the researcher. The researcher must check in with the buddy before and after a visit finishes or the buddy will follow escalation procedures. Check-lists provide guidance on what to do before and during the visits, for example ensuring phones are fully charged and being prepared to leave in an emergency if there are concerns about safety. A phrase is provided to enable the researcher to report an emergency during the visit. Guidance is provided for general safe travelling, for example to keep to well-lit paths and driveways.

DISSEMINATION

The results of this study will be communicated in relevant academic and professional journals, conferences and workshops, and via websites and social media, ensuring reach to all stakeholders (people living with the condition, professionals, commissioners and academics). A short film will be created to illustrate experiences of participation from the perspectives of people living with the condition, and those involved in delivery of the intervention. The manualised intervention will be refined and made available on the website in an open access format and it is anticipated that this may be of interest to both primary care and memory services.

STUDY STATUS

At the time of submitting for publication the study was collecting data. At the time of publication the study is approaching database lock.

AUTHOR CONTRIBUTIONS

GM, AF, CCo, KS, EL, SW, TY, CCr, EMC, AL, TD and KB co-wrote the original Trial Protocol. JW led the development of the Protocol for publication. JBD and KS developed the sections on fidelity and qualitative analysis. JBD developed the section on PPI. BDT adapted the introductory section, BJT the methods section, EY the outcomes section and EL the statistical analysis section. All authors contributed to reviewing and revising the draft versions prior to submission.

ACKNOWLEDGEMENTS

For peer review only - http://bmjopen.bmj.com/site/about/guidelines.xhtml

We would like to thank the following people for assisting with the original project application: Martin Orrell, Institute of Mental Health, The University of Nottingham; Daniel Blackburn, Sheffield Institute for Translational Neuroscience, The University of Sheffield; and Diana Papaioannou, Sheffield Clinical Trials Research Unit, School of Health and Related Research, The University of Sheffield.

Additionally, we would like to thank Peter Bowie, Old Age Psychiatry, Sheffield Health and Social Care NHS Foundation Trust, Sheffield, for advising on clinical aspects; and Nicholas Bell, Sheffield Health and Social Care NHS Foundation Trust, for acting on behalf of the sponsor and advising on trial procedures.

FUNDING STATEMENT

This study was funded by the NIHR Health Technology Assessment Programme (project number 14/140/80). The views expressed are those of the author(s) and not necessarily those of the NHS, the NIHR or the Department of Health.

COMPETING INTERESTS

All authors, with the exception of those below, declare no competing interests.

- SW, TY, CCo, JW, AF, AL, EL, BJT, BDT, EY as ScHARR has contracts and/or research grants with the Department of Health, NIHR, MRC and NICE.
- SW, CCo and TY are co-applicants or co-investigators on NIHR portfolio grants (NIHR Research Design Service for Yorkshire and Humber; HTA, RfPB, PHR; SDO) and grants from the MRC.
- SW and TY also receive external examining fees from various UK higher education institutes.
- SW receives book royalties from publishers including John Wiley and Sons Ltd and Blackwell Publishing.
- BDT is a Consultant for Arch research in the area of dementia.

ETHICS APPROVAL

This study was approved by Leeds East Research Ethics Committee, on 01/07/16, reference number 16/YH/0238. Health Research Authority approval was provided for the study to commence on 25/08/16. The current Protocol version is v7, 5th December 2018.

REFERENCES

1 World Health Organisation. The global burden of disease: 2004 update. Geneva: World Health Organisation 2008.

2 Knapp M, Comas-Herrera A, Somani A, *et al.* Dementia: international comparisons – summary report to the National Audit Office. London: London School of Economics 2007.

3 Department of Health. Prime Minister’s challenge on dementia 2020. London: Department of Health 2015. https://www.gov.uk/government/uploads/system/uploads/attachment_data/file/406076/Dementia_vision.pdf (accessed 26 Sep 2018)

For peer review only - http://bmjopen.bmj.com/site/about/guidelines.xhtml

4 Department of Health. Living well with dementia : A National Dementia Strategy. London: Department of Health 2009. https://www.gov.uk/government/publications/living-well-with-dementia-a-national-dementia-strategy (accessed 26 Sep 2018)

5 Hodge AS, Hailey E. English National Memory Clinics Audit Report. London: Royal College of Psychiatrists 2013. http://www.rcpsych.ac.uk/pdf/English National Memory Clinics Audit Report 2013.pdf (accessed 30 Oct 2018)

6 Banerjee S, Wittenberg R. Clinical and cost effectiveness of services for early diagnosis and intervention in dementia. *Int J Geriatr Psychiatry* 2009;24:748–54. doi:10.1002/gps.2191

7 Copland E, Hodge S, Clary L, *et al.* Memory Services National Accreditation Programme (MSNAP): Standards for memory services. London: Royal College of Psychiatrists 2018. https://www.rcpsych.ac.uk/pdf/MSNAP_Standards_6_Edition_2018.pdf (accessed 16 Oct 2018)

8 NHS England. Well Pathway for Dementia. 2017. https://www.england.nhs.uk/mentalhealth/wp-content/uploads/sites/29/2016/03/dementia-well-pathway.pdf (accessed 30 Oct 2018)

9 National Institute for Health and Clinical Excellence. Donepezil, galantamine, rivastigmine, and memantine for the treatment of Alzheimer's Disease. London: NIHCE 2011. http://www.nice.org.uk/guidance/ta217/resources/guidance-donepezilgalantaminerivastigmineandmemantine-for-the-treatment-of-alzheimers-disease-pdf (accessed 26 Sep 2018)

10 Woods B, Aguirre E, Spector AE, *et al.* Cognitive stimulation to improve cognitive functioning in people with dementia. *Cochrane Database Syst Rev* 2012;15. doi:10.1002/14651858.CD005562.pub2

11 Moniz-Cook E, Vernooij-Dassen M, Woods B, *et al.* Psychosocial interventions in dementia care research: The INTERDEM manifesto. *Aging Ment Health* 2011;15:283–90. doi:10.1080/13607863.2010.543665

12 McDermott O, Charlesworth G, Hogervorst E, *et al.* Psychosocial interventions for people with dementia: a synthesis of systematic reviews. *Aging Ment Health* Published Online First: 2018. doi:10.1080/13607863.2017.1423031

13 Clare L, Woods RT. Cognitive training and cognitive rehabilitation for people with early-stage Alzheimer's disease: A review. *Neuropsychol Rehabil* 2004;14:385–401. doi:10.1080/09602010443000074

14 Graff MJL, Vernooij-Dassen MJM, Thijssen M, *et al.* Community based occupational therapy for patients with dementia and their care givers: randomised controlled trial. *BMJ* 2006;333:1196. doi:10.1136/bmj.39001.688843.BE

15 Vernooij-Dassen M, Olde Rikkert MG. Personal disease management in dementia care. *Int J Geriatr Psychiatry* 2004;19:715–7. doi:10.1002/gps.1149

16 Moniz-Cook E, Manthorpe J, eds. Early Psychosocial Interventions in Dementia. London: Jessica Kingsley Publishers 2008.

17 Quinn C, Toms G, Anderson D, *et al.* A review of self-management interventions for people with dementia and mild cognitive impairment. *J. Appl. Gerontol.* 2016;35:1154–88. doi:10.1177/0733464814566852

For peer review only

18 Quinn C, Toms G, Jones C, *et al.* A pilot randomized controlled trial of a self-management group intervention for people with early-stage dementia (The SMART study). *Int Psychogeriatr* 2016;28:787–800. doi:10.1017/S1041610215002094

19 Martin F, Turner A, Wallace LM, *et al.* Qualitative evaluation of a self-management intervention for people in the early stage of dementia. *Dementia* 2015;14:418–35. doi:10.1177/1471301213498387

20 Clarke CL, Keyes SE, Wilkinson H, *et al.* Healthbridge: The National Evaluation of Peer Support Networks and Dementia Advisers in Implementation of the National Dementia Strategy for England. London: Department of Health 2013.

21 Chakkalackal L, Kalathil J. Evaluation report: Peer support groups to facilitate self-help coping strategies for people with dementia in extra care housing. London: Mental Health Foundation 2014.

22 Sprange K, Mountain GA, Shortland K, *et al.* Journeying through Dementia, a community-based self-management intervention for people aged 65 years and over: a feasibility study to inform a future trial. *Pilot Feasibility Stud* 2015;1:42. doi:10.1186/s40814-015-0039-6

23 Bellg AJ, Resnick B, Minicucci DS, *et al.* Enhancing treatment fidelity in health behavior change studies: Best practices and recommendations from the NIH Behavior Change Consortium. *Health Psychology* 2004;23:443–51. doi:10.1037/0278-6133.23.5.443

24 National Institute for Health and Care Excellence. Behaviour Change at population, community and individual levels. Public Health Guidance 6. London: NIHCE 2007.

25 Craig P, Dieppe P, Macintyre S, *et al.* Developing and evaluating complex interventions: new guidance. London: Medical Research Council 2008. http://www.mrc.ac.uk/Utilities/Documentrecord/index.htm?d=MRC004871 (Accessed 28 Sep 2018)

26 ISRCTN Registry. ISRCTN17993825 Journeying through dementia: exploring the clinical and cost-effectiveness of a group self-management intervention for people in the early stages of dementia. 2019. http://www.isrctn.com/ISRCTN17993825 (accessed 15 Jan 2019)

27 Alzheimer's Disease International. Participation in dementia trials and studies: Challenges and recommendations. 2014. https://www.alz.co.uk/sites/default/files/pdfs/dementia-trials.pdf (accessed 28 Sept 2018)

28 Herschell AD, Kolko DJ, Baumann BL, *et al.* The role of therapist training in the implementation of psychosocial treatments: A review and critique with recommendations. *Clin Psychol Rev* 2010;30:448–66. doi:10.1016/j.cpr.2010.02.005

29 Mountain GA, Craig CL. What should be in a self-management programme for people with early dementia? *Aging Ment Health* 2012;16:576–83. doi:10.1080/13607863.2011.651430

30 Mulhern B, Rowen D, Brazier J, *et al.* Development of DEMQOL-U and DEMQOL-PROXY-U: Generation of preference-based indices from DEMQOL and DEMQOL-PROXY for use in economic evaluation. *Health Technol Assess (Rockv)* 2013;17:1–140. doi:10.3310/hta17050

31 Smith S, Lamping D, Banerjee S, *et al.* Measurement of health-related quality of life for people with dementia: development of a new instrument (DEMQOL) and an evaluation of current methodology. *Health Technol Assess (Rockv)* 2005;9:1–93. doi:https://doi.org/10.3310/hta9100

For peer review only - http://bmjopen.bmj.com/site/about/guidelines.xhtml

32 Herdman M, Gudex C, Lloyd A, *et al.* Development and preliminary testing of the new five-level version of EQ-5D (EQ-5D-5L). *Qual Life Res* 2011;20:1727–36. doi:10.1007/s11136-011-9903-x

33 Hounsome N, Orrell M, Edwards RT. EQ-5D as a quality of life measure in people with dementia and their carers: Evidence and key issues. *Value Health* 2011;14:390–9. doi:10.1016/j.jval.2010.08.002

34 Spitzer RL, Williams JBW, Kroenke K. Test Review: Patient Health Questionnaire-9 (PHQ-9). *Rehabil Couns Bull* 2014;57:246–8. doi:10.1177/0034355213515305

35 Kroenke K, Spitzer RL, Williams JBW. The PHQ-9: Validity of a Brief Depression Severity Measure. *J Gen Intern Med* 2001;16:606–13. doi:10.1046/j.1525-1497.2001.016009606.x

36 Löwe B, Decker O, Müller S, *et al.* Validation and standardization of the generalized anxiety disorder screener (GAD-7) in the general population. *Medical Care* 2008;46:266–74. doi:10.1097/MLR.0b013e318160d093

37 Schwarzer R, Jerusalem M. Generalized Self-Efficacy scale. In: Weinman J, Wright S, Johnston M, eds. Measures in health psychology: A user's portfolio. Causal and control beliefs. Windsor: NFER-NELSON 1995. 35–7.

38 Diener E, Wirtz D, Tov W, *et al.* New measures of well-being: Flourishing and positive and negative feelings. In: Diener E, ed. Social Indicators Research Series. New York: Springer Science + Business Media 2009. 247–66.

39 De Vries R, Ryan KA, Stanczyk A, *et al.* Public's approach to surrogate consent for dementia research: Cautious pragmatism. *Am J Geriatr Psychiatry* 2013;21:364–72. doi:10.1016/j.jagp.2012.11.010

40 Lawton MP, Brody EM. Assessment of Older People : Self-Maintaining and Instrumental Activities of Daily Living 1. *Gerontologist* 1969;9:179–86. doi:10.1093/geront/9.3_Part_1.179

41 Vernooij-Dassen MJFJ, Persoon JMG, Felling AJA. Predictors of sense of competence in caregivers of demented persons. *Soc Sci Med* 1996;43:41–9. doi:10.1016/0277-9536(95)00332-0

42 Jansen APD, van Hout HPJ, van Marwijk HWJ, *et al.* Sense of competence questionnaire among informal caregivers of older adults with dementia symptoms: A psychometric evaluation. *Clin Pract Epidemiol Ment Health* 2007;3:1–11. doi:10.1186/1745-0179-3-11

43 Moniz-Cook E, Vernooij-Dassen M, Woods R, *et al.* A European consensus on outcome measures for psychosocial intervention research in dementia care. *Aging Ment Heal* 2008;1:29. doi:10.1080/13607860801919850

44 Schulz KF, Altman DG, Moher D. CONSORT 2010 Statement : updated guidelines for reporting parallel group randomised trials. *BMJ* 2010;340:332. doi:10.1136/bmj.c332

45 Baldwin SA, Bauer DJ, Stice E, *et al.* Evaluating models for partially clustered designs. *Psychol Methods* 2011;16:149–65. doi:10.1037/a0023464

46 Walters SJ. Therapist effects in randomised controlled trials: What to do about them. *J Clin Nurs* 2010;19:1102–12. doi:10.1111/j.1365-2702.2009.03067.x

47 Roberts C, Batistatou E, Roberts SA. Design and analysis of trials with a partially nested design

For peer review only

and a binary outcome measure. *Stat Med* 2015;35:1616–36. doi:10.1002/sim.6828

48 Kahan BC, Morris TP. Improper analysis of trials randomised using stratified blocks or minimisation. *Stat Med* 2012;31:328–40. doi:10.1002/sim.4431

49 NHS Improvement. Reference Costs 2017/18. 2018. https://improvement.nhs.uk/resources/reference-costs/ (accessed 15 Jan 2019)

50 Curtis L, Burns A. Unit Costs of Health & Social Care 2018. Canterbury: University of Kent 2018.

51 Schafer JL. Multiple imputation: A primer. *Stat Methods Med Res* 1999;8:3–15. doi:10.1191/096228099671525676

52 Van Hout BA, Al MJ, Gordon GS, *et al.* Costs, effects and C/E-ratios alongside a clinical trial. *Health Econ* 1994;3:309–19. doi:10.1002/hec.4730030505

53 McHugh ML. Interrater reliability: the kappa statistic. *Biochem Med (Zagreb)* 2012;22:276–82. doi:10.11613/BM.2012.031

54 Sprange K., Mountain GA, Brazier J, *et al.* Lifestyle Matters for maintenance of health and wellbeing in people aged 65 years and over: study protocol for a randomised controlled trial. *Trials* 2013;14:302. doi:10.1186/1745-6215-14-302

55 Ritchie J, Spencer L. Qualitative Data Analysis for Applied Policy Research. In: Humberman AM, Miles MB, eds. The Qualitative Researcher's Companion. London: SAGE Publications 2002. 305–29. doi:10.4135/9781412986274

56 Cooper C, Ketley D, Livingston G. Systematic review and meta-analysis to estimate potential recruitment to dementia intervention studies. *Int J Geriatr Psychiatry* 2014;29:515–25. doi:10.1002/gps.4034

57 HRA. GDPR guidance for researchers and study coordinators. London: Health Research Authority (HRA) 2019. https://www.hra.nhs.uk/planning-and-improving-research/policies-standards-legislation/data-protection-and-information-governance/gdpr-guidance/ (accessed 18 Jun 2019)

FIGURE LEGENDS

Figure 1: Participant flow through the study

For peer review only

For peer review only - http://bmjopen.bmj.com/site/about/guidelines.xhtml

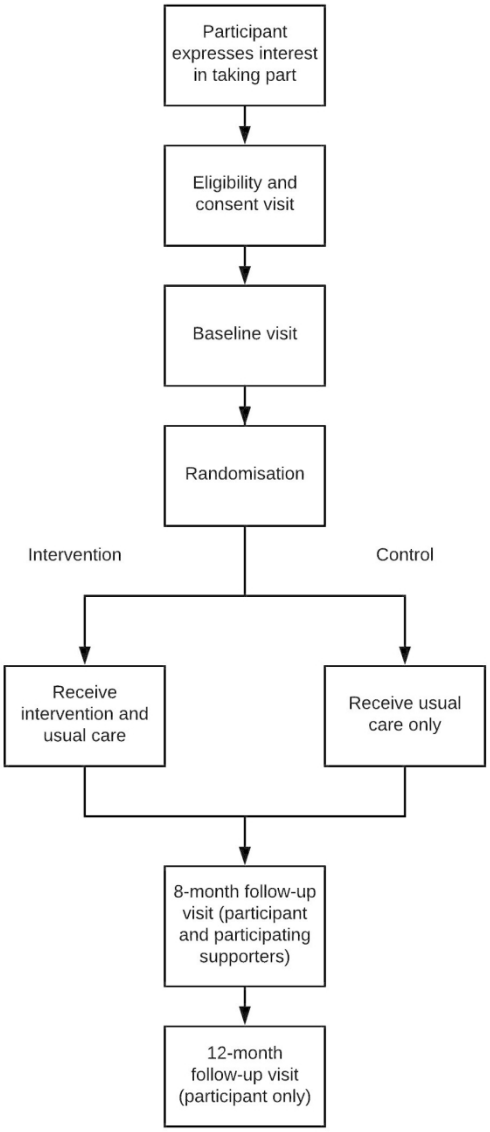

Figure 1 Participant flow through the study

92x190mm (300 x 300 DPI)

STANDARD PROTOCOL ITEMS: RECOMMENDATIONS FOR INTERVENTIONAL TRIALS

SPIRIT 2013 Checklist: Recommended items to address in a clinical trial protocol and related documents*

Section/item	Item No	Description	Addressed on page number
**Administrative information**			
Title	1	Descriptive title identifying the study design, population, interventions, and, if applicable, trial acronym	_________i____
Trial registration	2a	Trial identifier and registry name. If not yet registered, name of intended registry	________2_____
	2b	All items from the World Health Organization Trial Registration Data Set	____See 2a_____
Protocol version	3	Date and version identifier	________14______
Funding	4	Sources and types of financial, material, and other support	_____14______
Roles and responsibilities	5a	Names, affiliations, and roles of protocol contributors	______i-ii_______
	5b	Name and contact information for the trial sponsor	____13_______
	5c	Role of study sponsor and funders, if any, in study design; collection, management, analysis, and interpretation of data; writing of the report; and the decision to submit the report for publication, including whether they will have ultimate authority over any of these activities	_____n/a_______
	5d	Composition, roles, and responsibilities of the coordinating centre, steering committee, endpoint adjudication committee, data management team, and other individuals or groups overseeing the trial, if applicable (see Item 21a for data monitoring committee)	_____13________

For peer review only

Introduction

Background and rationale	6a	Description of research question and justification for undertaking the trial, including summary of relevant studies (published and unpublished) examining benefits and harms for each intervention	____2-3________
	6b	Explanation for choice of comparators	____2-3________
Objectives	7	Specific objectives or hypotheses	____3_________
Trial design	8	Description of trial design including type of trial (eg, parallel group, crossover, factorial, single group), allocation ratio, and framework (eg, superiority, equivalence, noninferiority, exploratory)	____3-4________

Methods: Participants, interventions, and outcomes

Study setting	9	Description of study settings (eg, community clinic, academic hospital) and list of countries where data will be collected. Reference to where list of study sites can be obtained	______5_______
Eligibility criteria	10	Inclusion and exclusion criteria for participants. If applicable, eligibility criteria for study centres and individuals who will perform the interventions (eg, surgeons, psychotherapists)	____4-5________
Interventions	11a	Interventions for each group with sufficient detail to allow replication, including how and when they will be administered	____5-6____
	11b	Criteria for discontinuing or modifying allocated interventions for a given trial participant (eg, drug dose change in response to harms, participant request, or improving/worsening disease)	________n/a_____
	11c	Strategies to improve adherence to intervention protocols, and any procedures for monitoring adherence (eg, drug tablet return, laboratory tests)	______8-9_____
	11d	Relevant concomitant care and interventions that are permitted or prohibited during the trial	______6_____
Outcomes	12	Primary, secondary, and other outcomes, including the specific measurement variable (eg, systolic blood pressure), analysis metric (eg, change from baseline, final value, time to event), method of aggregation (eg, median, proportion), and time point for each outcome. Explanation of the clinical relevance of chosen efficacy and harm outcomes is strongly recommended	_____6-7__
Participant timeline	13	Time schedule of enrolment, interventions (including any run-ins and washouts), assessments, and visits for participants. A schematic diagram is highly recommended (see Figure)	____Table 1 and figure 1_____

Sample size	14	Estimated number of participants needed to achieve study objectives and how it was determined, including clinical and statistical assumptions supporting any sample size calculations	___9______
Recruitment	15	Strategies for achieving adequate participant enrolment to reach target sample size	_____5_______

Methods: Assignment of interventions (for controlled trials)

Allocation:

Sequence generation	16a	Method of generating the allocation sequence (eg, computer-generated random numbers), and list of any factors for stratification. To reduce predictability of a random sequence, details of any planned restriction (eg, blocking) should be provided in a separate document that is unavailable to those who enrol participants or assign interventions	_____4________
Allocation concealment mechanism	16b	Mechanism of implementing the allocation sequence (eg, central telephone; sequentially numbered, opaque, sealed envelopes), describing any steps to conceal the sequence until interventions are assigned	____4_________
Implementation	16c	Who will generate the allocation sequence, who will enrol participants, and who will assign participants to interventions	____4_______
Blinding (masking)	17a	Who will be blinded after assignment to interventions (eg, trial participants, care providers, outcome assessors, data analysts), and how	___4______
	17b	If blinded, circumstances under which unblinding is permissible, and procedure for revealing a participant's allocated intervention during the trial	______4_______

Methods: Data collection, management, and analysis

Data collection methods	18a	Plans for assessment and collection of outcome, baseline, and other trial data, including any related processes to promote data quality (eg, duplicate measurements, training of assessors) and a description of study instruments (eg, questionnaires, laboratory tests) along with their reliability and validity, if known. Reference to where data collection forms can be found, if not in the protocol	____6-8_______
	18b	Plans to promote participant retention and complete follow-up, including list of any outcome data to be collected for participants who discontinue or deviate from intervention protocols	____6-7_______

For peer review only - http://bmjopen.bmj.com/site/about/guidelines.xhtml

Data management	19	Plans for data entry, coding, security, and storage, including any related processes to promote data quality (eg, double data entry; range checks for data values). Reference to where details of data management procedures can be found, if not in the protocol	__12
Statistical methods	20a	Statistical methods for analysing primary and secondary outcomes. Reference to where other details of the statistical analysis plan can be found, if not in the protocol	____9-11_
	20b	Methods for any additional analyses (eg, subgroup and adjusted analyses)	__11_
	20c	Definition of analysis population relating to protocol non-adherence (eg, as randomised analysis), and any statistical methods to handle missing data (eg, multiple imputation)	_10-11___
**Methods: Monitoring**			
Data monitoring	21a	Composition of data monitoring committee (DMC); summary of its role and reporting structure; statement of whether it is independent from the sponsor and competing interests; and reference to where further details about its charter can be found, if not in the protocol. Alternatively, an explanation of why a DMC is not needed	____13_____
	21b	Description of any interim analyses and stopping guidelines, including who will have access to these interim results and make the final decision to terminate the trial	______n/a______
Harms	22	Plans for collecting, assessing, reporting, and managing solicited and spontaneously reported adverse events and other unintended effects of trial interventions or trial conduct	___12-13____
Auditing	23	Frequency and procedures for auditing trial conduct, if any, and whether the process will be independent from investigators and the sponsor	____12______
**Ethics and dissemination**			
Research ethics approval	24	Plans for seeking research ethics committee/institutional review board (REC/IRB) approval	______14_______
Protocol amendments	25	Plans for communicating important protocol modifications (eg, changes to eligibility criteria, outcomes, analyses) to relevant parties (eg, investigators, REC/IRBs, trial participants, trial registries, journals, regulators)	_______13______

For peer review only - http://bmjopen.bmj.com/site/about/guidelines.xhtml

Consent or assent	26a	Who will obtain informed consent or assent from potential trial participants or authorised surrogates, and how (see Item 32)	______5______
	26b	Additional consent provisions for collection and use of participant data and biological specimens in ancillary studies, if applicable	__See supplementary information__
Confidentiality	27	How personal information about potential and enrolled participants will be collected, shared, and maintained in order to protect confidentiality before, during, and after the trial	___12-13_____
Declaration of interests	28	Financial and other competing interests for principal investigators for the overall trial and each study site	_____14______
Access to data	29	Statement of who will have access to the final trial dataset, and disclosure of contractual agreements that limit such access for investigators	_____12____
Ancillary and post-trial care	30	Provisions, if any, for ancillary and post-trial care, and for compensation to those who suffer harm from trial participation	_____n/a______
Dissemination policy	31a	Plans for investigators and sponsor to communicate trial results to participants, healthcare professionals, the public, and other relevant groups (eg, via publication, reporting in results databases, or other data sharing arrangements), including any publication restrictions	_____13___
	31b	Authorship eligibility guidelines and any intended use of professional writers	______n/a______
	31c	Plans, if any, for granting public access to the full protocol, participant-level dataset, and statistical code	______12______
**Appendices**			
Informed consent materials	32	Model consent form and other related documentation given to participants and authorised surrogates	See supplementary materials_____
Biological specimens	33	Plans for collection, laboratory evaluation, and storage of biological specimens for genetic or molecular analysis in the current trial and for future use in ancillary studies, if applicable	_____n/a________

For peer review only - http://bmjopen.bmj.com/site/about/guidelines.xhtml

*It is strongly recommended that this checklist be read in conjunction with the SPIRIT 2013 Explanation & Elaboration for important clarification on the items. Amendments to the protocol should be tracked and dated. The SPIRIT checklist is copyrighted by the SPIRIT Group under the Creative Commons "Attribution-NonCommercial-NoDerivs 3.0 Unported" license.

For peer review only

For peer review only - http://bmjopen.bmj.com/site/about/guidelines.xhtml
